# Health-Related Quality of Life in Long-Term Prostate Cancer Survivors Who Received Hormone Therapy: A Scoping Review

**DOI:** 10.3390/curroncol33030137

**Published:** 2026-02-26

**Authors:** Maya Basbous, Keyi Yang, Volker Arndt, Melissa S. Y. Thong

**Affiliations:** 1 Unit of Cancer Survivorship, German Cancer Research Center (DKFZ), 69120 Heidelberg, Germany; maya.basbous@dkfz-heidelberg.de (M.B.); yangkeyi1234@hotmail.com (K.Y.); v.arndt@dkfz-heidelberg.de (V.A.); 2 Medical Faculty of Heidelberg, Heidelberg University, 69120 Heidelberg, Germany

**Keywords:** prostate cancer, androgen deprivation therapy, hormone therapy, health-related quality of life

## Abstract

Prostate cancer accounts for the largest group of cancer survivors in men. Hormone therapy is essential, especially in advanced disease. While its short-term effects are well studied, research into the long-term effects on health-related quality of life (HRQOL) remains limited. Therefore, this review aims to synthesize and identify key gaps in the literature on HRQOL and symptom burden in long-term prostate cancer survivors who underwent hormone therapy. After searching four databases until 15 April 2025, we identified 14 observational studies that reported on general and prostate cancer-specific HRQOL in prostate cancer survivors ≥ 5 years post-diagnosis. Survivors who underwent hormone therapy reported worse global health status and physical, emotional, and social functioning compared to those treated with local therapies like prostatectomy or radiation. These survivors also experienced greater symptom burdens, alongside worse vitality and mental health. Prostate cancer-specific issues, such as bowel and urinary bother and sexual dysfunction, were also more pronounced in hormone therapy recipients. Nevertheless, outcomes beyond 15 years remain under-researched. The findings of this review highlight the importance of discussing long-term HRQOL compromises with patients who consider hormone therapy. However, they warrant cautious interpretation, particularly due to limited details on hormone therapy regimens and inadequate control for stage.

## Simple Summary

Many men now live for many years after prostate cancer diagnosis. Hormone therapy is one of the main treatments available, especially when the cancer is advanced, but its long-term effects on daily life are not well understood. In this review, we looked at studies of men who survived at least 5 years after prostate cancer diagnosis and had received hormone therapy. The findings show that, compared with men treated only with local therapies, such as surgery or radiation, those who had hormone therapy often reported poorer overall health, more physical and emotional difficulties, lower energy, and greater problems with social life. They also experienced more bowel, urinary, and sexual problems. In addition, very little research has followed men beyond 15 years. These insights highlight the need for clearer discussions with patients about long-term effects and for future studies to better understand how different hormone treatments affect long-term quality of life.

## 1. Introduction

Prostate cancer is the second most common cancer in men worldwide, with over 1.4 million new cases and more than 390,000 deaths each year [1,2]. The number of new cases of prostate cancer is predicted to double by 2040, due to population aging, increased life expectancy, and increasing rates of prostate-specific antigen (PSA) testing [3]. Advances in early diagnosis and treatment modalities have significantly improved prognosis, achieving a 5-year relative survival rate of 97.5% [4]. In addition, mortality from prostate cancer has decreased in most Western countries [5,6]. This has led to a growing population of long-term prostate cancer survivors, defined as those living 5 years or more after diagnosis [7].

Treatment options for prostate cancer include active surveillance, surgery, radiation therapy, hormone therapy, chemotherapy, immunotherapy, and targeted therapy, with the choice depending on the stage and aggressiveness of the disease [8]. Hormone therapy includes traditional androgen deprivation therapy (ADT), such as LHRH agonists and antagonists, as well as first- and second-generation anti-androgens, which bind to androgen receptors to prevent androgens from stimulating tumor growth [9]. Due to the significant androgen dependence of prostate cancer cells, ADT has been considered the cornerstone of systemic treatment for men with prostate cancer since 1941 [10]. One of the benefits of ADT is its ability to effectively reduce levels of circulating testosterones, which fuel the growth of prostate cancer cells [11]. Prostate cancer patients with advanced and recurrent disease often require systemic treatment, including ADT [12]. ADT is also increasingly used either as monotherapy or in combination with other treatment modalities for the management of clinically localized prostate cancer, and is often prescribed as a lifelong treatment [13,14]. Despite its efficacy in controlling disease progression, ADT has been associated with several adverse effects. These include cardiovascular complications, osteoporosis, impaired cognitive function, sexual dysfunction, fatigue, and reduced sleep quality. Certain side effects are specifically associated with the use of second-generation antiandrogens, such as hypertension and hypothyroidism [11]. These side effects can persist long after treatment, affecting multiple aspects of survivors’ health and health-related quality of life (HRQOL) [15]. Moreover, most of these symptoms are chronic in nature, which raises concerns about the long-term impact on prostate cancer survivors who undergo hormone therapy [15].

As hormone therapy becomes more broadly indicated, and an increasing number of prostate cancer survivors are maintained on long-term prescriptions, it is imperative to investigate the long-term effects of hormone therapy on the HRQOL, including physical and psychological wellbeing [16,17,18]. Consistent with established oncological definitions, long-term survivorship is defined as 5 or more years after diagnosis [19]. Several studies have explored the HRQOL of prostate cancer survivors treated with hormone therapy, particularly physical health, mental health, and sexual functioning [15,17,18,20]. However, most of these studies have been conducted on prostate cancer patients during or shortly after their treatment, with only few studies having examined the long-term HRQOL of prostate cancer survivors [15]. In addition, previous systematic reviews evaluating HRQOL in prostate cancer patients receiving hormone therapy have primarily focused on comparisons between different hormone therapy regimens rather than assessing the independent long-term impact of hormone therapy on overall quality of life [21,22]. These reviews have also concentrated on individual symptoms (e.g., erectile dysfunction, gynecomastia, etc.) rather than domain-specific HRQOL outcomes, often without applying a predefined follow-up threshold, with most included studies assessing patients within 5 years of diagnosis [21,22]. Hence, there is a need to explore the long-term HRQOL across its different domains. The purpose of this scoping review is therefore to synthesize and identify key gaps in the available literature on HRQOL in long-term prostate cancer survivors who have been treated with hormone therapy. The clinical relevance of HRQOL in long-term prostate cancer survivors is heightened by the expanding indications for hormone therapy and the increasing life expectancy of affected men, resulting in prolonged exposure to hormone treatment-related adverse effects. Consequently, understanding long-term, domain-specific HRQOL outcomes is essential for balancing hormone treatment benefits against impact on quality of life.

## 2. Materials and Methods

We used the Preferred Reporting Items for Systematic Reviews and Meta-Analyses extension for Scoping Reviews (PRISMA-ScR) checklist to report the review findings (Supplementary Materials, Table S1) [23]. The protocol for this scoping review is publicly accessible online on the Open Science Framework: https://osf.io/jn5xg [24].

### 2.1. Information Sources and Search Strategy

The first author (MB) developed a comprehensive search strategy and searched four databases (MEDLINE (OVID), Embase.com, CINAHL (EBSCO), and Cochrane). Tables S2–S5 in the Supplementary Materials provide the search strategy used in the aforementioned databases. The search was limited to articles published in English language, with no restrictions on date or study type. We used MeSH terms and relevant keywords related to prostate cancer, hormone therapy and HRQOL. We reviewed articles published up to 15 April 2025. An expert librarian (AH) at DKFZ reviewed and verified the search strategy. We also scrutinized the reference lists of eligible studies to retrieve potentially relevant publications. All included studies were imported and stored in EndNote, with duplicates removed, where applicable.

### 2.2. Eligibility Criteria

Study selection criteria for this scoping review are as follows:

Inclusion criteria:

- Peer-reviewed, published original research articles (observational and randomized controlled trials);
- Studies reporting on survivors of prostate cancer who are either at least 5 years post-diagnosis, or have been followed-up for 5 or more years post-diagnosis;
- Studies reporting on the HRQOL of prostate cancer survivors who received hormone therapy, either alone or combined with other treatment modalities.

Exclusion criteria:

- Commentaries, editorials, case reports, case series, conference abstracts, and reviews;
- Non-English articles;
- Studies in which both comparison groups receive some form of hormone therapy, either alone or in combination with other treatment modalities.

### 2.3. Outcomes

Our primary outcome was general HRQOL. Our secondary outcome was prostate cancer-specific HRQOL and symptom burden.

### 2.4. Study Selection and Evidence Abstraction

Two reviewers (MB, KY) screened titles and abstracts of all identified records. Subsequently, both reviewers screened full texts of potentially eligible articles, utilizing an a priori developed full-text screening sheet (Supplementary File S1). This process was carried out independently and in duplicate to ensure a thorough and unbiased assessment. Any exclusions made were documented, including the reasons for exclusion. We used the software Rayyan [25] for recording the full texts of screening decisions.

Using standardized extraction forms, both reviewers extracted data from all included records independently and in duplicate. Extracted data included the author’s name, country and year of publication, study design, population, patients’ demographics, sample size, type and duration of hormone treatment, other treatment modalities, HRQOL instrument, covariates and results, if available.

Any disagreements during these stages were resolved through discussion or the help of a third reviewer, who is an expert in the field (MT or VA).

### 2.5. Risk of Bias Assessment

While not required for scoping reviews, two reviewers (MB, KY) independently assessed the methodological quality and risk of bias of each included observational study, using the Joanna Briggs Institute critical appraisal checklists for observational studies to guide cautious interpretation of the findings [26]. Each question was rated as ‘yes’, ‘no’, ‘unclear’, or ‘not applicable’. We calculated the percentage of ‘yes’ responses for each study, and categorized the risk of bias as follows: low (>80%), moderate (51–80%), and high (≤50%).

### 2.6. Data Synthesis

Given the variability in outcome measures and years after diagnosis of prostate cancer, we summarized the findings of this review using a descriptive approach, emphasizing common patterns across studies. The results from the included studies were summarized in a table, and grouped by outcomes and corresponding HRQOL instruments.

## 3. Results

**Figure 1:**
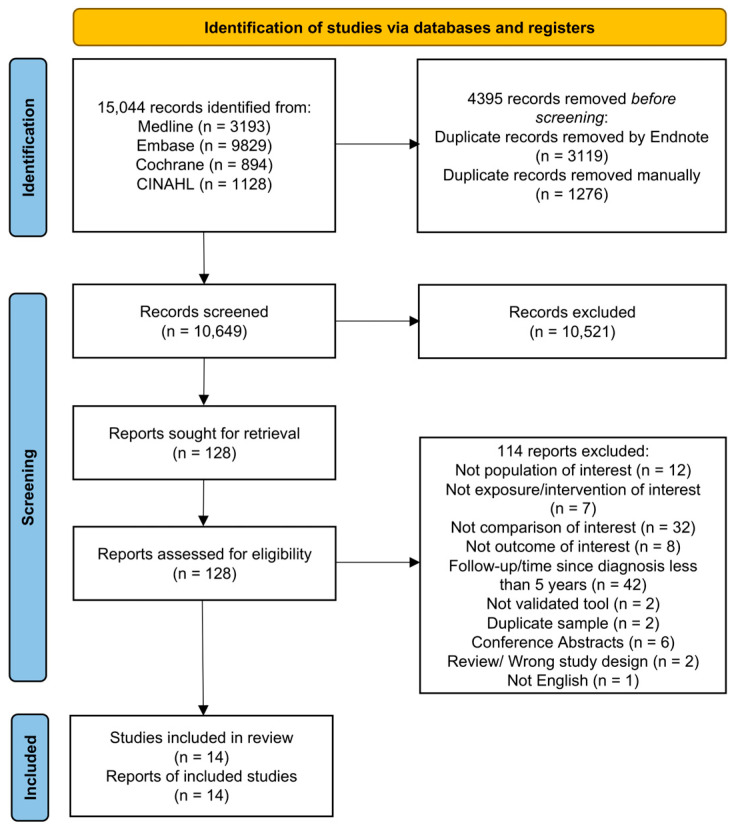
Flow diagram of articles related to use of hormone therapy and quality of life of prostate cancer survivors [27].

The search strategy identified 15,044 citations. Following duplicate removal, we screened 10,649 citations, of which 128 articles were potentially eligible. From the 128 articles, we retained a total of 14 articles (Figure 1), and excluded 114 articles for the reasons outlined in Table S6 (Supplementary Materials). All of the included articles were observational studies that reported on the HRQOL of prostate cancer survivors. We did not identify any eligible clinical trials.

### 3.1. Description of Included Studies

Table S9 (Supplementary Materials) describes the characteristics of the 14 included observational studies, listed in descending chronological order from 2024 to 2002 [28,29,30,31,32,33,34,35,36,37,38,39,40,41]. These studies reported on general HRQOL (*n* = 13), prostate cancer-specific HRQOL (*n* = 12), and other psychological wellbeing issues (*n* = 2). The exposure was hormone therapy, either alone or in combination with other treatments, compared to one or more other treatment modalities. Nine studies included only ADT in their comparisons [28,30,32,33,35,36,37,38,41], while the remaining studies included any type of hormone therapy, including both classical ADT and anti-androgens [29,31,34,39,40]. The included studies were conducted in Europe (*n* = 8), North America (*n* = 5), and Australia (*n* = 1). Prostate cancer patients were recruited from population-based cancer registries (*n* = 7), national databases (*n* = 2), hospitals (*n* = 1), urology clinics (*n* = 3), and using a convenience sample (*n* = 1). The sample size ranged from 143 to 6944. Five studies were cohort, six were cross-sectional, and three were a combination of cross-sectional and case–control designs. Participants were followed from 6 months post-diagnosis up to 19.8 years after end of treatment in cohort studies. In cross-sectional studies, prostate cancer patients/survivors were 5 to 18 years post-diagnosis. In addition to survivors more than 5 years post-diagnosis, three cross-sectional studies also included survivors who were less than 5 years post-diagnosis [29,38,40].

Thirteen studies on general HRQOL used various questionnaires [28,29,30,31,32,33,34,35,37,38,39,40,41] (Table 1). Six of them measured general HRQOL using the European Organization for Research and Treatment of Cancer Core Quality of Life Questionnaire (EORTC QLQ-C30) [31,33,34,35,38,40], while the remaining studies used the 12-Item Short Form Health Survey (SF-12) [28,30,32], the 36-Item Short Form Health Survey (SF-36) [28,37,39], Functional Assessment of Cancer Therapy-General (FACT-G) [29], Quality of Life Cancer Survivors Questionnaire (QoL-CS) [39], or a questionnaire based on SF-12 and published literature [41].

Eleven of the 13 studies that reported general HRQOL also reported prostate cancer-specific HRQOL and symptoms, in addition to one other study [28,29,30,32,33,34,35,36,37,38,40,41] (Table 2). Four studies used the European Organization for Research and Treatment of Cancer Prostate Cancer 25 Questionnaire (EORTC QLQ-PR25) [33,34,38,40]. The remaining studies used the Expanded Prostate Cancer Index Composite (EPIC) questionnaire [28,32,35], the University of California Los Angeles Prostate Cancer Index (UCLA PCI) [30,37], the Functional Assessment of Cancer Therapy-General (FACT-P) [29], and the Prostate Cancer Symptom Scale (PCSS)-based questionnaire [36].

Tables S7 and S8 (Supplementary Materials) provide the detailed assessment of the risk of bias and the overall appraisal for each of the included studies. In the assessment of the cross-sectional studies, six were classified as having a low risk of bias, demonstrating adherence to rigorous methodological standards [31,33,35,36,38,39]. Two studies were assigned a moderate risk of bias, primarily due to their reliance on self-reported treatment modalities and adjustment for age as the only confounder, without accounting for other factors such as cancer stage [40,41]. Another study exhibited a high risk of bias, with significant concerns related to self-reporting of treatment and insufficient control for confounding variables [29]. Regarding the cohort studies, three were classified as having a low risk of bias, meeting robust methodological criteria [28,32,37]. One study was deemed to have a moderate risk of bias, primarily due to loss to follow-up and inadequate strategies to address incomplete follow-up [30]. The final cohort study exhibited a high risk of bias, with notable issues concerning incomplete follow-up and failure to adequately identify and control for confounding variables, significantly compromising the integrity of its findings [34].

### 3.2. Association of Hormone Therapy with HRQOL

#### 3.2.1. General HRQOL–Functioning

Prostate cancer survivors with various treatment combinations, involving hormone therapy, reported worse HRQOL outcomes, including global health status/QOL, physical functioning, emotional functioning, social functioning, and physical and mental wellbeing, compared to survivors who received local treatment alone (e.g., RP and/or RT) or those who were in active surveillance/watchful waiting. One study compared the HRQOL of prostate cancer survivors who survived 2 to 18 years after diagnosis and received ADT exclusively to those who underwent radical prostatectomy [38]. Similarly, the analysis showed that worse global health status/QOL, physical functioning, role functioning, and social functioning were observed in the ADT group.

Two studies included primary hormone therapy in their comparisons. Punnen et al. reported a decline in physical health 5 years after diagnosis in prostate cancer survivors who received primary ADT compared to nerve-sparing radical prostatectomy (adjusted OR = 1.9, 95% CI [1.1–2.2]; *p*-value = 0.03) [37]. The second study, by Mols et al., also showed that primary hormone therapy was associated with worse physical functioning (adjusted mean score = 57.4 (31.8)) compared to radical prostatectomy (adjusted mean score = 75.7 (24.4); *p*-value < 0.05) and other treatment combinations that could also involve hormone therapy (RP ± RT ± HT), in prostate cancer patients who survived 5 to 10 years after diagnosis [39]. Vitality was also worse in the primary hormone therapy group (adjusted mean score = 62.1 (22.4)) compared to radical prostatectomy (adjusted mean score = 70.9 (20.8); *p* < 0.05) and other treatment combinations involving hormone therapy. Moreover, the authors of that study assessed generic HRQOL survival issues using QOL-CS, and also observed worse physical and psychological well-being in prostate cancer survivors who received primary hormone therapy.

Finally, one study compared medical to surgical castration and reported a lower mental score with time in the medical castration group [30]. Another study assessed the effect of castration, either medical or surgical, on the HRQOL of prostate cancer survivors 7 to 8 years after prostatectomy. Survivors in the castration group reported greater prostate cancer- and treatment-related concerns, including body image concerns, and worries about cancer and death, and had lower scores in general health, mental health and activity index [41].

Only one study showed no difference in the general HRQOL of survivors of localized prostate cancer who received ADT compared to those who did not, 10 years after diagnosis [35].

#### 3.2.2. General HRQOL—Symptom Burden

Prostate cancer survivors who received hormone therapy also reported higher burden of fatigue, dyspnea, nausea and vomiting, insomnia, diarrhea, appetite loss, and financial difficulties (Table 1).

#### 3.2.3. Prostate Cancer-Specific HRQOL

Prostate cancer survivors who received hormone therapy with various other combinations had worse bowel symptoms and function, hormone treatment-related symptoms and function, urinary bother, particularly burning on urination, and sexual function than survivors who received no hormone therapy or who were observed only (Table 2). Survivors 2 to 18 years after diagnosis who received ADT exclusively reported less sexual activity and more ADT-related symptoms, compared with those who had radical prostatectomy [38]. One study reported worse urinary incontinence, urinary and bowel bother, and sexual function in survivors who received ADT, with or without external beam radiation therapy, at both 10 and 15 years post-diagnosis, compared to age-matched controls [32]. Particularly, urinary incontinence and urinary bother worsened between year 10 and year 15, whereas sexual function showed improvement during the same period. Similarly, another study compared ADT as a single therapy and in combination with either radiotherapy or radical prostatectomy, or both, with age-matched controls [36]. Survivors who received ADT alone reported more erectile dysfunction compared with controls (adjusted OR = 2.32, 95% CI [1.33–4.05]). In addition, survivors who received ADT in combination with other forms of therapy had worse erectile and bowel dysfunction and urinary urgency and incontinence. The only study comparing primary hormone therapy to radical prostatectomy showed a decline in bowel function (adjusted OR = 2, 95% CI [1.1–3.6]; *p*-value = 0.03) and bother (adjusted OR = 2, 95% CI [1.1–3.8]; *p*-value = 0.03) 5 years after diagnosis [37].

In addition, one study showed that medical castration was associated with worse urinary and hormone function, and bowel bother, compared to surgical castration [30]. Finally, one study reported on self-treatment side effects of castration in prostate cancer survivors 7 to 8 years after prostatectomy. Survivors in the castration group reported worse sexual function and significantly fewer days with sexual drive [41].

#### 3.2.4. Psychological Well-Being Issues

Evidence on psychological outcomes is limited, as only two studies explored psychological issues related to prostate cancer survivors on hormone treatment [29,35]. One used a cross-sectional design, and one was a combination of cross-sectional and case–control designs. There was substantial heterogeneity across these studies in terms of measurement tools and the psychological outcomes evaluated. The authors found no differences in depression, anxiety, or psychological flexibility between long-term prostate cancer survivors who underwent hormone therapy and those who did not. However, one study showed that prostate cancer patients who survived an average of 5 years and who received any form of hormone therapy reported lower masculine self-esteem compared with those who did not receive any (Table 3).

## 4. Discussion

Hormone therapy in prostate cancer is associated with worse general and prostate cancer-specific HRQOL outcomes in long-term survivors, i.e., 5 and more years after diagnosis/treatment. Studies using validated questionnaires revealed that survivors who underwent hormone therapy reported worse global health status and physical, emotional, and social functioning compared to those treated with local therapies like radical prostatectomy or radiation. These survivors also experienced greater symptom burdens, alongside worse vitality and mental health. Prostate cancer-specific issues, such as bowel and urinary bother and sexual dysfunction, were also more pronounced in hormone therapy recipients. While psychological well-being outcomes like depression and anxiety showed no differences according to hormone therapy status, lower masculine self-esteem was noted in survivors on hormone therapy.

This review builds upon previous literature examining hormone therapy and HRQOL in prostate cancer patients by specifically focusing on long-term survivors. While earlier studies have reported that ADT is associated with reduced HRQOL, alongside increased emotional and cognitive impairment, depressive symptoms, and fatigue, they often did not distinguish between short- and long-term survivors [42,43]. Despite evidence that newer antiandrogens can delay HRQOL deterioration and reduced pain progression, they also reported increased fatigue and hot flushes [43,44]. However, emotional well-being was generally unaffected [44]. Our findings align with some of these results, but provide a more detailed perspective on HRQOL in long-term survivors.

To our knowledge, our scoping review is the first to systematically map domain-specific HRQOL outcomes in long-term prostate cancer survivors who received hormone therapy, using a predefined follow-up threshold (≥5 years after diagnosis). Unlike previous studies, which primarily focused on early survivorship [43], our approach enables a clearer understanding of which specific dimensions of HRQOL remain affected in long-term survivors who received hormone therapy, thus providing clinicians with a domain-specific overview of persistent HRQOL impairments.

Due to the scarcity of longitudinal studies that have tracked prostate cancer survivors for more than 5 years post-diagnosis, only a limited number of studies were eligible for inclusion in this review. Most of included studies focused on prostate cancer survivors up to 15 years post-diagnosis, with little evidence available on HRQOL beyond 15 years. Additionally, the majority of studies in the existing literature have explored primarily the effect of local treatment options, like radical prostatectomy and radiation therapy, with little focus on hormone therapy. Moreover, the variation in how prostate cancer stage was reported and adjusted for across studies highlights an important methodological gap. While some studies specified disease stage [28,30,32,33,34,35,36,37] or adjusted for it as a covariate [38,39], others included all stages without adjustment [29,40] or did not report stage at all [31,41]. When comparing studies that did not report or adjust for stage with those that did, studies adjusting for stage reported similar results but showed additional burden in social functioning, appetite loss, psychological wellbeing, vitality, and bowel and urinary symptoms. This inconsistency in accounting for a key clinical characteristic like stage limits the ability to draw firm conclusions regarding the independent impact of disease stage on long-term HRQOL and underscores the need for more standardized reporting in future research. Furthermore, there is a lack of detailed information regarding hormone therapy regimens and their duration, and whether they were administered intermittently or continuously, which further complicates the analysis. A systematic review and meta-analysis comparing intermittent to continuous ADT showed that most included trials reported an improvement in physical and sexual functioning with intermittent therapy [45]. Contrasting results were highlighted in a review by Luo and collaborators, which found different effects on HRQOL and symptoms burden among recipients of the new generation of antiandrogens [43]. Therefore, this review cannot determine whether particular hormone therapy regimens or treatment durations are associated with differential long-term HRQOL effects. In addition, the disparity in HRQOL measurement tools and differences in follow-up duration introduced further challenges to the analysis. On the other hand, using a range of HRQOL questionnaires captures the full spectrum of relevant domains, enhancing generalizability by incorporating aspects assessed in some questionnaires but absent in others. In our review, we included only two studies that specifically examined primary hormone therapy [37,39], whereas the others involved combined treatment modalities, which complicates further the comparison across studies. While hormone therapy is a crucial treatment for prostate cancer, its use varies based on stage, patient characteristics and treatment goals [46]. Primary hormone therapy is often used as the main treatment for patients who are not candidates for local therapy, typically in advanced diseases, and its goal is systemic control and symptom management [46]. However, hormone therapy in combination with surgery or radiation therapy is usually administered to target microscopic residual disease [46]. Patients receiving adjuvant or neoadjuvant hormone therapy often have earlier-stage disease but adverse pathological features [46]. These differences highlight the importance of tailoring comparisons to specific patients’ characteristics and treatment goals.

## 5. Conclusions

By applying a scoping methodology, we were able to provide a structured overview of methodological challenges, which provides a clear agenda for future research studies. To summarize, the findings of this review highlight the importance of discussing HRQOL compromises with patients who consider hormone therapy for prostate cancer. These discussions should consider cancer stage and clinical characteristics, as they can influence the decision to initiate hormone therapy and its impact on HRQOL. Future research studies should also prioritize longitudinal designs to evaluate the trajectory of HRQOL changes over time in long-term prostate cancer survivors and explore interventions to improve HRQOL for recipients of hormone therapy.

## Figures and Tables

**Table 1:** Summary of general HRQOL and symptom burden in the included studies.

Questionnaire	Author	Comparison	Burden in the Group Including Hormone Therapy
EORTC QLQ-C30	Jackson et al. [31]	HT ± EBRT vs. no HT ^1^	Physical functioning, fatigue, dyspnea, financial difficulties
	Adam et al. [33]	ADT (±RP and RT) vs. RP vs. RT vs. RP and RT vs. NTX ^1^	Nausea and vomiting
		ADT (±RP and RT) vs. RP ^1^	Global health status/QOL, emotional functioning, social functioning, fatigue, nausea and vomiting, dyspnea, insomnia, diarrhea, financial difficulties
		ADT (±RP and RT) vs. RT ^1^	Nausea and vomiting, dyspnea, insomnia, financial difficulties
		ADT (±RP and RT) vs. NTX ^1^	Emotional functioning, fatigue, nausea and vomiting, dyspnea, financial difficulties
	Hammerer et al. [34]	ADT (±AA or RT or other TX) vs. controls	Physical functioning
	Kerleau et al. [35]	ADT vs. no ADT ^1^	-
	Drummond et al. [38]	ADT alone vs. ADT + EBRT vs. EBRT alone vs. RP vs. BT vs. observation	Global health status, physical functioning, role functioning, cognitive functioning, social functioning, fatigue, nausea and vomiting, pain, dyspnea, insomnia, appetite loss, constipation
		ADT alone vs. RP ^1^	Global health status, physical functioning, role functioning, social functioning, fatigue, pain, dyspnea, appetite loss
		ADT + EBRT vs. RP ^1^	-
	Voerman et al. [40]	HT vs. RP vs. RT vs. RP and RT vs. WW	General QOL, physical functioning, role functioning, fatigue, pain, insomnia
		HT vs. RP	Physical functioning
		HT vs. RT	General QOL
		HT vs. RP and RT	General QOL, physical functioning, role functioning, fatigue, pain
		HT vs. WW	General QOL, role functioning, fatigue, pain, insomnia
		HT vs. RP vs. RT vs. RP and RT vs. WW ^1^	General QOL, physical functioning
SF-12	Al Hussein et al. [28]	EBRT + ADT vs. RP	Physical health at year 10
		EBRT + ADT vs. RP ^1^	-
	Gaither et al. [30]	Medical vs. surgical castration	-
		Medical vs. surgical castration ^1^	Mental composite score with time
	Mazariego et al. [32]	ADT ± EBRT vs. controls ^1^	Physical wellbeing at year 15, mental wellbeing at year 15
SF-36	Al Hussein et al. [28]	EBRT + ADT vs. RP	Physical functioning at year 5
		EBRT + ADT vs. RP ^1^	-
	Punnen et al. [37]	Primary ADT vs. NSRP ^1,2^	Decline in physical health from 0 to 5 years
	Mols et al. [39]	Primary HT vs. RP ± HT ± RT vs. RT ± HT vs. WW ^1^	Physical functioning, vitality, social functioning, physical component scale
		Primary HT vs. RP ± HT ± RT ^1^	Physical functioning, vitality
QoL-CS	Mols et al. [39]	Primary HT vs. RP ± HT ± RT vs. RT ± HT vs. WW ^1^	Physical wellbeing, psychological wellbeing
FACT-G	Chowdhury et al. [29]	HT vs. no HT	QOL
Questionnaire based on SF-12 and previous studies	Fowler et al. [41]	ADT or orchiectomy (currently or not currently) ± RP vs. no ADT ^1^	Impact of cancer and treatment, concern about body image, mental health, general health, activity, worries about cancer and dying, energy

^1^ Adjusted for age ± other covariates; ^2^ Using imputed values. Abbreviations: AA: Antiandrogens; ADT: Androgen Deprivation Therapy; BT: Brachytherapy; EBRT: External Beam Radiation Therapy; EORTC QLQ-C30: European Organization for Research and Treatment of Cancer Quality of Life questionnaire; FACT-G: Functional Assessment of Cancer Therapy-General; HT: Hormone Treatment; HRQOL: Health-Related Quality of Life; NSRP: Nerve-Sparing Radical Prostatectomy; NTX: No Treatment Reported; QOL: Quality of Life; QoL-CS: Quality of Life Cancer Survivors; RP: Radical Prostatectomy; RT: Radiation Therapy; SF-12: The 12-Item Short Form Health Survey; SF-36: The 36-Item Short Form Health Survey; TX: Treatment; WW: Watchful Waiting.

**Table 2:** Summary of prostate-cancer specific HRQOL in the included studies.

Questionnaire	Author	Comparison	Burden in the Group Including Hormone Therapy
EPIC/EPIC-26	Al Hussein et al. [28]	EBRT + ADT vs. RP	Burning on urination at year 10 (higher in EBRT + ADT), bowel function at years 5 and 10, lack of energy at year 10 (higher in EBRT + ADT)
		EBRT + ADT vs. RP ^1^	Burning on urination at year 10 (higher in EBRT + ADT), bowel function at year 10, hormone function at year 10
	Mazariego et al. [32]	ADT ± EBRT vs. controls ^1^	Urinary incontinence at year 15 (worsening from year 10 to 15), urinary bother at year 15 (significant worsening from year 10 to 15), sexual function at years 5, 10 and 15 (improvement from year 5 to 15), bowel bother at years 5, 10 and 15 (minimal worsening from year 5 to 15)
	Kerleau et al. [35]	ADT vs. no ADT ^1^	Sexual function, hormonal problems
FACT-P	Chowdhury et al. [29]	HT vs. no HT	Prostate cancer symptoms
UCLA PCI	Gaither et al. [30]	Medical vs. surgical castration	Sexual function (higher score in medical castration group)
		Medical vs. surgical castration ^1^	Urinary function, bowel bother (with time), hormone function (through time)
	Punnen et al. [37]	Primary ADT vs. NSRP ^1,2^	Decline in bowel function and bowel bother from 0 to 5 years
EORTC QLQ-PR25	Adam et al. [33]	ADT (±RP and RT) vs. RP vs. RT vs. RP and RT vs. NTX ^1^	Bowel symptoms, HT-related symptoms, sexual activity
		ADT (±RP and RT) vs. RP ^1^	Bowel symptoms, HT-related symptoms, sexual activity
		ADT (±RP and RT) vs. RT ^1^	HT-related symptoms, sexual activity
		ADT (±RP and RT) vs. NTX ^1^	Urinary bother, bowel symptoms, HT-related symptoms, sexual activity
	Hammerer et al. [34]	ADT (±AA or RT or other TX) vs. controls	Sexual activity, sexual functioning, HT-related symptoms
	Drummond et al. [38]	ADT alone vs. ADT + EBRT vs. EBRT alone vs. RP vs. BT vs. observation	Urinary symptoms, bowel symptoms (highest in ADT + EBRT), ADT-related symptoms, sexual activity (highest in ADT)
		ADT alone vs. RP ^1^	ADT-related symptoms, sexual activity
		ADT + EBRT vs. RP ^1^	Bowel symptoms, ADT-treatment symptoms, sexual activity
	Voerman et al. [40]	HT vs. RP vs. RT vs. RP and RT vs. WW	Bowel problems, HT-related problems, sexual functioning
		HT vs. RP	HT-related problems, sexual functioning
		HT vs. RT	HT-related problems, sexual functioning
		HT vs. RP and RT	HT-related problems, sexual functioning
		HT vs. WW	HT-related problems, sexual functioning
		HT vs. RP vs. RT vs. RP and RT vs. WW ^2^	HT-related problems, sexual functioning
PCSS-based questionnaire	Carlsson et al. [36]	ADT alone vs. controls ^1^	Erectile dysfunction
		ADT + RP vs. controls ^1^	Erectile dysfunction, urinary incontinence
		ADT + RT vs. controls ^1^	Erectile dysfunction, urinary urgency, bowel dysfunction
		ADT + RP + RT vs. controls ^1^	Erectile dysfunction, urinary incontinence, bowel dysfunction
Self-reported side effects	Fowler et al. [41]	ADT or orchiectomy (currently or not currently) ± RP vs. no ADT ^1^	Sexual function, days felt sexual drive in past 30 days

^1^ Adjusted for age ± other covariates; ^2^ Using imputed values. Abbreviations: AA: Antiandrogens; ADT: Androgen Deprivation Therapy; BT: Brachytherapy; EBRT: External Beam Radiation Therapy; EORTC QLQ-PR25: European Organization for Research and Treatment of Cancer Quality of Life Questionnaire-Prostate 25; EPIC: Expanded Prostate Cancer Index Composite; FACT-P: Functional Assessment of Cancer Therapy-Prostate; HT: Hormone Treatment; HRQOL: Health-Related Quality of Life; NSRP: Nerve-Sparing Radical Prostatectomy; NTX: No Treatment Reported; PCSS: Prostate Cancer Symptom Scale; RP: Radical Prostatectomy; RT: Radiation Therapy; TX: Treatment; UCLA PCI: University of California Los Angeles Prostate Cancer Index; WW: Watchful Waiting.

**Table 3:** Summary of psychological wellbeing issues in the included studies.

Questionnaire	Author	Comparison	Burden in the Group Including Hormone Therapy
DASS-21	Chowdhury et al. [29]	HT vs. no HT	-
MSE Scale	Chowdhury et al. [29]	HT vs. no HT	Masculine self esteem
CompACT processes measures	Chowdhury et al. [29]	HT vs. no HT	-
HADS	Kerleau et al. [35]	ADT vs. no ADT ^1^	-

^1^ Adjusted for age ± other covariates. Abbreviations: ADT: Androgen Deprivation Therapy; CompACT: Comprehensive Assessment of Acceptance and Commitment Therapy; DASS-21: Depression, Anxiety, and Stress Scale-21 items; HADS: Hospital Anxiety and Depression Scale; HT: Hormone Treatment; MSE: Masculine Self Esteem.

## Data Availability

No new data were created in this study.

## Supplementary Materials

The following supporting information can be downloaded at: https://www.mdpi.com/article/10.3390/curroncol33030137/s1, Table S1. Preferred Reporting Items for Systematic reviews and Meta-Analyses extension for Scoping Reviews (PRISMA-ScR) Checklist. Table S2. Medline Search Strategy. Table S3. Embase Search Strategy. Table S4. Cochrane Search Strategy. Table S5. CINAHL Search Strategy. Table S6. Reasons for exclusion. Table S7. Risk of bias and quality assessment of included cross-sectional studies using the Joanna Briggs Institute (JBI) critical appraisal tool. Table S8. Risk of bias and quality assessment of included cohort studies using the Joanna Briggs Institute (JBI) critical appraisal tool. Table S9. Characteristics of the included studies. Supplementary File S1. Full-text screening form.

## Abbreviations

The following abbreviations are used in this manuscript:

AA	Antiandrogens
ADT	Androgen Deprivation Therapy
AS	Active Surveillance
BT	Brachytherapy
CI	Confidence Interval
CompACT	Comprehensive Assessment of Acceptance and Commitment Therapy
DASS-21	Depression, Anxiety, and Stress Scale-21 items
EBRT	External Beam Radiation Therapy
EORTC QLQ-C30	European Organization for Research and Treatment of Cancer Quality of Life-Core 30
EORTC QLQ-PR25	European Organization for Research and Treatment of Cancer Quality of Life-Prostate 25
EPIC	Expanded Prostate Cancer Index Composite
FACT-G	Functional Assessment of Cancer Therapy-General
FACT-P	Functional Assessment of Cancer Therapy-Prostate
HADS	Hospital Anxiety and Depression Scale
HRQOL	Health-Related Quality of Life
HT	Hormone Therapy
IQR	Interquartile Range
LHRH	Luteinizing Hormone Releasing Hormone
MeSH	Medical Subject Heading
MSE	Masculine Self Esteem
NSRP	Nerve-Sparing Radical Prostatectomy
NTX	No Treatment Reported
OR	Odds Ratio
PC	Prostate Cancer
PCSS	Prostate Cancer Symptom Scale
PRISMA-ScR	Preferred Reporting Items for Systematic Reviews and Meta-Analyses extension for Scoping Reviews
PSA	Prostate Specific Antigen
QOL	Quality of Life
QoL-CS	Quality of Life-Cancer Survivors
RP	Radical Prostatectomy
RT	Radiation Therapy
SD	Standard Deviation
SF-12	The 12-Item Short Form Health Survey
SF-36	The 36-Item Short Form Health Survey
TX	Treatment
UCLA PCI	University of California Los Angeles Prostate Cancer Index
WW	Watchful Waiting
