# Supplementary material for: Health-Related Quality of Life in Long-Term Prostate Cancer Survivors Who Received Hormone Therapy: A Scoping Review
Source: Curr Oncol. 2026 Feb 26;33(3):137. doi: 10.3390/curroncol33030137 (PMC13024926; doi:10.3390/curroncol33030137)
Supplement: Supplementary file 1 [file curroncol-33-00137-s001.zip › curroncol-4137988-supplementary.pdf]

Supplementary Materials

Table S1. Preferred Reporting Items for Systematic reviews and Meta-Analyses extension for Scoping Reviews (PRISMA-ScR) Checklist 2

Table S2. Medline Search Strategy (Database: Ovid MEDLINE(R) and Epub Ahead of Print, In-Process, In-Data-Review & Other Non-Indexed Citations, Daily and Versions <1946 to April 14, 2025>) 4

Table S3. Embase Search Strategy (Date Run: 15 April 2025) 7

Table S4. Cochrane Search Strategy (Date Run: 15/04/2025) 11

Table S5. CINAHL Search Strategy 14

Supplementary File S1. Full-text screening form 19

Table S6. Reasons for exclusion 20

Table S7. Risk of bias and quality assessment of included cross-sectional studies using the Joanna Briggs Institute (JBI) critical appraisal tool 23

Table S8. Risk of bias and quality assessment of included cohort studies using the Joanna Briggs Institute (JBI) critical appraisal tool 24

Table S9. Characteristics of the included studies 25

References 29

Table S1. Preferred Reporting Items for Systematic reviews and Meta-Analyses extension for Scoping Reviews (PRISMA-ScR) Checklist [1]

SECTION	ITEM	PRISMA-ScR CHECKLIST ITEM	REPORTED ON PAGE #
		**TITLE**	
**Title**	1	Identify the report as a scoping review.	1
		**ABSTRACT**	
**Structured summary**	2	Provide a structured summary that includes (as applicable): background, objectives, eligibility criteria, sources of evidence, charting methods, results, and conclusions that relate to the review questions and objectives.	1
		**INTRODUCTION**	
**Rationale**	3	Describe the rationale for the review in the context of what is already known. Explain why the review questions/objectives lend themselves to a scoping review approach.	2
**Objectives**	4	Provide an explicit statement of the questions and objectives being addressed with reference to their key elements (e.g., population or participants, concepts, and context) or other relevant key elements used to conceptualize the review questions and/or objectives.	2
		**METHODS**	
**Protocol and registration**	5	Indicate whether a review protocol exists; state if and where it can be accessed (e.g., a Web address); and if available, provide registration information, including the registration number.	3
**Eligibility criteria**	6	Specify characteristics of the sources of evidence used as eligibility criteria (e.g., years considered, language, and publication status), and provide a rationale.	3
**Information sources***	7	Describe all information sources in the search (e.g., databases with dates of coverage and contact with authors to identify additional sources), as well as the date the most recent search was executed.	3
**Search**	8	Present the full electronic search strategy for at least 1 database, including any limits used, such that it could be repeated.	Supplementary Materials Tables S2-S5
**Selection of sources of evidence**	9	State the process for selecting sources of evidence (i.e., screening and eligibility) included in the scoping review.	3
**Data charting process**	10	Describe the methods of charting data from the included sources of evidence (e.g., calibrated forms or forms that have been tested by the team before their use, and whether	3

		data charting was done independently or in duplicate) and any processes for obtaining and confirming data from investigators.	
**Data items**	11	List and define all variables for which data were sought and any assumptions and simplifications made.	3
**Critical appraisal of individual sources of evidence**	12	If done, provide a rationale for conducting a critical appraisal of included sources of evidence; describe the methods used and how this information was used in any data synthesis (if appropriate).	3-4
**Synthesis of results**	13	Describe the methods of handling and summarizing the data that were charted.	4
		**RESULTS**	
**Selection of sources of evidence**	14	Give numbers of sources of evidence screened, assessed for eligibility, and included in the review, with reasons for exclusions at each stage, ideally using a flow diagram.	4 and Supplementary Materials Table S6
**Characteristics of sources of evidence**	15	For each source of evidence, present characteristics for which data were charted and provide the citations.	5 and Supplementary Materials Table S9
**Critical appraisal within sources of evidence**	16	If done, present data on critical appraisal of included sources of evidence (see item 12).	6 and Supplementary Materials Tables S7-8
**Results of individual sources of evidence**	17	For each included source of evidence, present the relevant data that were charted that relate to the review questions and objectives.	5-7, 9
**Synthesis of results**	18	Summarize and/or present the charting results as they relate to the review questions and objectives.	8-9
		**DISCUSSION**	
**Summary of evidence**	19	Summarize the main results (including an overview of concepts, themes, and types of evidence available), link to the review questions and objectives, and consider the relevance to key groups.	9
**Limitations**	20	Discuss the limitations of the scoping review process.	10
**Conclusions**	21	Provide a general interpretation of the results with respect to the review questions and objectives, as well as potential implications and/or next steps.	11
		**FUNDING**	
**Funding**	22	Describe sources of funding for the included sources of evidence, as well as sources of funding for the scoping review. Describe the role of the funders of the scoping review.	11

Table S2. Medline Search Strategy (Database: Ovid MEDLINE(R) and Epub Ahead of Print, In-Process, In-Data-Review & Other Non-Indexed Citations, Daily and Versions <1946 to April 14, 2025>)

No.	Query	Results
**1**	exp prostatic neoplasms/	158,371
**2**	(prostat* adj3 (cancer* or carcin* or adenocarcin* or neoplas* or tumo?r* or anticarcinogen* or anti-carcionogen* or (anti adj carcinogen*) or malignan* or oncogen* or oncolog*))	214,503
**3**	1 or 2	214,503
**4**	exp androgen antagonists/	19,668
**5**	exp antineoplastic agents, hormonal/	189,246
**6**	gonadotropin-releasing hormone/ or buserelin/ or goserelin/ or leuprolide/ or triptorelin pamoate/	35,240
**7**	((androgen* adj3 (inhibit* or antagonis* or depriv* or blocker* or blockade*)) or (anti adj androgen*) or antiandrogen* or anti-androgen* or (androgen* adj2 receptor* adj2 signal* adj2 inhibit*) or arsi)	30,872
**8**	(hormon* adj3 (drug* or medication* or therap* or treatment* or agent*))	100,953
**9**	(((gonadotropin or gnrh or ((lute?nizing or lute?nising) adj1 hormone) or lhrh) adj3 (antagonist? or agonist? or analog*)) or (anti adj gonadotropin*) or antigonadotropin* or anti-gonadotropin* or gonadorelin)	18,003
**10**	(goserelin or leuprolide or leuprorelin or leuprolin or leuprolidine or triptorelin or buserelin or etilamide or degarelix or abiraterone or bicalutamide or flutamide or niftolide or enzalutamide or apalutamide or darolutamide or ketodarolutamide or darramamide or cyproterone or aminoglutethimide or (chlormadinone adj acetate) or chlormadinone-acetate)	27,056
**11**	(zoladex or eligard or fensolvi or lupron or lucrin or viadur or camcevi or decapeptyl or trelstar or triptodur or suprefact or suprecur or firmagon or zytiga or yonsa or casodex or cosudex or calutide or calumid or kalumid or bicadex or bical or bicalox or bicamide or bicatlon or bicusan or binabic or bypro or calutol or ormandyl or eulexin or xtandi or erleada or nubeqa or androcur or androcur-100 or androstat or asoteron or cyprone or cyproplex or cyprostat or cysaxal or imvel or siterone or eliptenor or cytadren or orimeten or belara or gynorelle or luteran or prostal)	1,481
**12**	or/4-11	325,823
**13**	health status/ or functional status/ or psychosocial functioning/ or exp "quality of life"/ or "Activities of Daily Living"/	435,632
**14**	health care surveys/ or patient reported outcome measures/	52,453
**15**	((qualit* adj2 (life or living)) or qol or hrqol or (hr adj qol) or hr-qol or hrql or (hr adj ql) or hr-ql or hql or hqol or (h adj qol))	531,675
**16**	(qualit* adj2 (wellbeing or well-being or (well adj being)))	3,888
**17**	(health adj2 (level* or qualit* or state* or status or index or indices or function* or outcome* or profil*))	654,740
**18**	(patient* adj2 satisfaction).mp.	131,970
**19**	((patient* adj2 outcome* adj measure*) or (patient-report* adj outcome* adj measure*) or (self-report* adj2 outcome*) or PRO or PROM or PROMs or hrpro or hr-pro or (hr adj pro) or hrprom or hr-prom or (hr adj prom) or hrproms or hr-proms or (hr adj proms))	340,098

No.	Query	Results
**20**	(sf36 or sf-36 or (sf adj "36") or (short adj form adj "36") or (shortform adj "36") or (short-form adj "36") or (sf adj thirtysix) or (sf adj thirty adj six) or (sf adj thirty-six) or (shortform adj thirtysix) or (shortform adj thirty adj six) or (shortform adj thirty-six) or (short adj form adj thirty adj six) or (short adj form adj thirtysix) or (short adj form adj thirty-six))	33,414
**21**	(sf6 or sf-6 or (sf adj "6") or (short adj form adj "6") or (shortform adj "6") or (short-form adj "6") or (sf adj six) or (shortform adj six) or (short adj form adj six) or (short-form adj six))	2,953
**22**	(sf12 or sf-12 or (sf adj "12") or (short adj form adj "12") or (shortform adj "12") or (short-form adj "12") or (sf adj twelve) or (shortform adj twelve) or (short adj form adj twelve) or (short-form adj twelve))	8,647
**23**	(sf16 or sf-16 or (sf adj "16") or (short adj form adj "16") or (shortform adj "16") or (short-form adj "16") or (sf adj sixteen) or (sf adj six adj teen) or (sf adj six-teen) or (shortform adj sixteen) or (shortform adj six adj teen) or (shortform adj six-teen) or (short adj form adj six adj teen) or (short adj form adj sixteen) or (short adj form adj six-teen))	42
**24**	(sf20 or sf-20 or (sf adj "20") or (short adj form adj "20") or (shortform adj "20") or (short-form adj "20") or (sf adj twenty) or (shortform adj twenty) or (short adj form adj twenty) or (short-form adj twenty))	480
**25**	(euroqol or (euro adj qol) or eq5d or (eq adj 5d) or eq-5d)	19,995
**26**	(flic or (functional adj living adj index adj cancer))	1,487
**27**	((functional adj assessment adj2 chronic adj illness adj therapy) or facit)	1,949
**28**	((quality adj2 life adj cancer adj survivor*) or (quality adj2 life-cancer adj survivor*) or qol-cs or (qol adj cs))	51
**29**	((quality adj2 life adj index) or qli or qli-cv or (qli adj cv))	5,837
**30**	((quality adj2 life adj2 adult adj cancer adj survivors) or ql-acs or qlacs or (ql adj acs))	51
**31**	((body adj image adj2 relationships adj scale) or birs)	56
**32**	((european adj organization adj2 research adj2 treatment adj2 cancer adj core adj "30") or (european adj organization adj2 research adj2 treatment adj2 cancer adj core-30) or (european adj organization adj2 research adj2 treatment adj2 cancer adj core30) or (european adj organization adj2 research adj2 treatment adj2 cancer adj c30) or (eortc adj qlq adj c adj "30") or (eortc adj qlq-c-30) or (eortc adj qlq-c30) or eortc-qlq-c-30 or eortc-qlq-c30 or (eortc adj core adj quality adj2 life) or (eortc adj core adj "30") or (eortc adj core30) or (eortc adj c30) or (european adj organization adj2 research adj2 treatment adj2 cancer adj prostate adj cancer adj "25") or (european adj organization adj2 research adj2 treatment adj2 cancer adj pr-25) or (european adj organization adj2 research adj2 treatment adj2 cancer adj pr25) or (european adj organization adj2 research adj2 treatment adj2 cancer adj pr adj "25") or (eortc adj qlq adj pr adj "25") or (eortc adj qlq-pr-25) or (eortc adj qlq-pr25) or eortc-qlq-pr-25 or eortc-qlq-pr25 or (eortc adj2 pr adj "25") or (eortc adj2 pr25))	5,107
**33**	(fact-g or fact-p or (functional adj assessment adj2 cancer adj therapy) or (functional adj assessment adj2 cancer adj therapy adj general) or (functional adj assessment adj2 cancer adj therapy-general) or (functional adj assessment adj2 cancer adj therapy adj prostate) or (functional adj assessment adj2 cancer adj therapy-prostate))	3,667
**34**	(escap-cdv or (escap adj cdv))	2
**35**	(epic or (expanded adj prostate adj cancer adj index))	6,613
**36**	(pc-qol or (pc adj qol) or (prostate adj cancer adj quality adj2 life adj instrument))	25

No.	Query	Results
**37**	(pcsi or (prostate adj cancer adj symptom adj indices))	144
**38**	(porpus or (patient-oriented adj prostate adj utility adj scale) or (patient adj oriented adj prostate adj utility adj scale))	23
**39**	(ucla-pci or (ucla adj pci) or (university adj2 california adj los adj angeles adj prostate adj cancer adj index) or (university adj2 california adj los adj angeles-prostate adj cancer adj index))	214
**40**	(WHOQOL-100 or WHO-QOL-100 or WHO-QOL100 or (WHOQOL adj "100") or (WHO adj QOL adj "100") or (WHO-QOL adj "100") or (WHO adj QOL100) or (WHO adj QOL-100) or (world adj health adj organi?ation adj quality adj2 life adj "100") or (world adj health adj organi?ation-quality adj2 life adj "100") or (world adj health adj organi?ation adj quality adj2 life-100) or (world adj health adj organi?ation-quality adj2 life-100) or WHOQOL-BREF or WHO-QOL-BREF or (WHOQOL adj BREF) or (WHO adj QOL adj BREF) or (WHO-QOL adj BREF) or (WHO adj QOL-BREF) or (world adj health adj organi?ation adj quality adj2 life adj BREF) or (world adj health adj organi?ation-quality adj2 life adj BREF) or (world adj health adj organi?ation adj quality adj2 life-BREF) or (world adj health adj organi?ation-quality adj2 life-BREF))	4,351
**41**	(IPSS or (international adj prostate adj symptom adj score))	8,049
**42**	or/13-41	1,566,657
**43**	3 and 12 and 42	3,485
**44**	limit 43 to english language	3,193

Table S3. Embase Search Strategy (Date Run: 15 April 2025)

No.	Query	Results
**#49**	#3 AND #15 AND #47 AND [english]/lim	9,829
**#48**	#3 AND #15 AND #47	10,282
**#47**	#16 OR #17 OR #18 OR #19 OR #20 OR #21 OR #22 OR #23 OR #24 OR #25 OR #26 OR #27 OR #28 OR #29 OR #30 OR #31 OR #32 OR #33 OR #34 OR #35 OR #36 OR #37 OR #38 OR #39 OR #40 OR #41 OR #42 OR #43 OR #44 OR #45 OR #46	2,391,927
**#46**	ipss OR (international NEXT/1 prostate NEXT/1 symptom NEXT/1 score)	21,992
**#45**	whoqol$100 OR who$qol$100 OR who$qol100 OR (whoqol NEXT/1 '100') OR (who NEXT/1 qol NEXT/1 '100') OR (who$qol NEXT/1 '100') OR (who NEXT/1 qol100) OR (who NEXT/1 qol$100) OR (world NEXT/1 health NEXT/1 organi?ation NEXT/1 quality NEAR/2 life NEXT/1 '100') OR (world NEXT/1 health NEXT/1 organi?ation$quality NEAR/2 life NEXT/1 '100') OR (world NEXT/1 health NEXT/1 organi?ation NEXT/1 quality NEAR/2 life$100) OR (world NEXT/1 health NEXT/1 organi?ation$quality NEAR/2 life$100) OR whoqol$bref OR who$qol$bref OR (whoqol NEXT/1 bref) OR (who NEXT/1 qol NEXT/1 bref) OR (who$qol NEXT/1 bref) OR (who NEXT/1 qol$bref) OR (world NEXT/1 health NEXT/1 organi?ation NEXT/1 quality NEAR/2 life NEXT/1 bref) OR (world NEXT/1 health NEXT/1 organi?ation$quality NEAR/2 life NEXT/1 bref) OR (world NEXT/1 health NEXT/1 organi?ation NEXT/1 quality NEAR/2 life$bref) OR (world NEXT/1 health NEXT/1 organi?ation$quality NEAR/2 life$bref)	7,567
**#44**	ucla$pci OR (ucla NEXT/1 pci) OR (university NEAR/2 california NEXT/1 los NEXT/1 angeles NEXT/1 prostate NEXT/1 cancer NEXT/1 index) OR (university NEAR/2 california NEXT/1 los NEXT/1 angeles$prostate NEXT/1 cancer NEXT/1 index)	330
**#43**	porpus OR (patient$oriented NEXT/1 prostate NEXT/1 utility NEXT/1 scale) OR (patient NEXT/1 oriented NEXT/1 prostate NEXT/1 utility NEXT/1 scale)	43
**#42**	pcsi OR (prostate NEXT/1 cancer NEXT/1 symptom NEXT/1 indices)	298
**#41**	pc$qol OR (pc NEXT/1 qol) OR (prostate NEXT/1 cancer NEXT/1 quality NEAR/2 life NEXT/1 instrument)	68
**#40**	epic OR (expanded NEXT/1 prostate NEXT/1 cancer NEXT/1 index)	16,668
**#39**	escap$cdv OR (escap NEXT/1 cdv)	3
**#38**	fact$g OR fact$p OR (functional NEXT/1 assessment NEAR/2 cancer NEXT/1 therapy) OR (functional NEXT/1 assessment NEAR/2 cancer NEXT/1 therapy NEXT/1 general) OR (functional NEXT/1 assessment NEAR/2 cancer NEXT/1 therapy$general) OR (functional NEXT/1 assessment NEAR/2 cancer NEXT/1 therapy NEXT/1 prostate) OR (functional NEXT/1 assessment NEAR/2 cancer NEXT/1 therapy$prostate)	7,792

No.	Query	Results
#37	(european NEXT/1 organization NEAR/2 research NEAR/2 treatment NEAR/2 cancer NEXT/1 core NEXT/1 '30') OR (european NEXT/1 organization NEAR/2 research NEAR/2 treatment NEAR/2 cancer NEXT/1 core$30) OR (european NEXT/1 organization NEAR/2 research NEAR/2 treatment NEAR/2 cancer NEXT/1 core30) OR (european NEXT/1 organization NEAR/2 research NEAR/2 treatment NEAR/2 cancer NEXT/1 c30) OR (eortc NEXT/1 qlq NEXT/1 c NEXT/1 '30') OR (eortc NEXT/1 qlq$c$30) OR (eortc NEXT/1 qlq$c30) OR eortc$qlq$c$30 OR eortcqlqc30 OR (eortc NEXT/1 core NEXT/1 quality NEAR/2 life) OR (eortc NEXT/1 core NEXT/1 '30') OR (eortc NEXT/1 core30) OR (eortc NEXT/1 c30) OR (european NEXT/1 organization NEAR/2 research NEAR/2 treatment NEAR/2 cancer NEXT/1 prostate NEXT/1 cancer NEXT/1 '25') OR (european NEXT/1 organization NEAR/2 research NEAR/2 treatment NEAR/2 cancer NEXT/1 pr$25) OR (european NEXT/1 organization NEAR/2 research NEAR/2 treatment NEAR/2 cancer NEXT/1 pr25) OR (european NEXT/1 organization NEAR/2 research NEAR/2 treatment NEAR/2 cancer NEXT/1 pr NEXT/1 '25') OR (eortc NEXT/1 qlq NEXT/1 pr NEXT/1 '25') OR (eortc NEXT/1 qlqpr25) OR (eortc NEXT/1 qlq$pr25) OR eortc$qlqpr25 OR eortcqlqpr25 OR (eortc NEAR/2 pr NEXT/1 '25') OR (eortc NEAR/2 pr25)	10,798
#36	(body NEXT/1 image NEAR/2 relationships NEXT/1 scale) OR birs	132
#35	(quality NEAR/2 life NEAR/2 adult NEXT/1 cancer NEXT/1 survivor*) OR ql$acs OR qlacs OR (ql NEXT/1 acs)	105
#34	(quality NEAR/2 life NEXT/1 index) OR qli OR qli$cv OR (qli NEXT/1 cv)	6,430
#33	(quality NEAR/2 life NEXT/1 cancer NEXT/1 survivor*) OR (quality NEAR/2 life$cancer NEXT/1 survivor*) OR qol$cs OR (qol NEXT/1 cs)	87
#32	(functional NEXT/1 assessment NEAR/2 chronic NEXT/1 illness) OR facit	5,237
#31	flic OR (functional NEXT/1 living NEXT/1 index NEXT/1 cancer)	1,820
#30	euroqol OR (euro NEXT/1 qol) OR eq5d OR (eq NEXT/1 5d) OR eq$5d	35,474
#29	sf20 OR sf$20 OR (sf NEXT/1 '20') OR (short NEXT/1 form NEXT/1 '20') OR (shortform NEXT/1 '20') OR (short$form NEXT/1 '20') OR (sf NEXT/1 twenty) OR (shortform NEXT/1 twenty) OR (short NEXT/1 form NEXT/1 twenty) OR (short$form NEXT/1 twenty)	699
#28	sf16 OR sf$16 OR (sf NEXT/1 '16') OR (short NEXT/1 form NEXT/1 '16') OR (shortform NEXT/1 '16') OR (short$form NEXT/1 '16') OR (sf NEXT/1 sixteen) OR (sf NEXT/1 six NEXT/1 teen) OR (sf NEXT/1 six$teen) OR (shortform NEXT/1 sixteen) OR (shortform NEXT/1 six NEXT/1 teen) OR (shortform NEXT/1 six$teen) OR (short NEXT/1 form NEXT/1 six NEXT/1 teen) OR (short NEXT/1 form NEXT/1 sixteen) OR (short NEXT/1 form NEXT/1 six$teen)	137
#27	sf12 OR sf$12 OR (sf NEXT/1 '12') OR (short NEXT/1 form NEXT/1 '12') OR (shortform NEXT/1 '12') OR (short$form NEXT/1 '12') OR (sf NEXT/1 twelve) OR (shortform NEXT/1 twelve) OR (short NEXT/1 form NEXT/1 twelve) OR (short$form NEXT/1 twelve)	17,046
#26	sf6 OR sf$6 OR (sf NEXT/1 '6') OR (short NEXT/1 form NEXT/1 '6') OR (shortform NEXT/1 '6') OR (short$form NEXT/1 '6') OR (sf NEXT/1 six) OR (shortform NEXT/1 six) OR (short NEXT/1 form NEXT/1 six) OR (short$form NEXT/1 six)	49,763

No.	Query	Results
#25	sf36 OR sf$36 OR (sf NEXT/1 '36') OR (short NEXT/1 form NEXT/1 '36') OR (shortform NEXT/1 '36') OR (short$form NEXT/1 '36') OR (sf NEXT/1 thirtysix) OR (sf NEXT/1 thirty NEXT/1 six) OR (sf NEXT/1 thirty$six) OR (shortform NEXT/1 thirtysix) OR (shortform NEXT/1 thirty NEXT/1 six) OR (shortform NEXT/1 thirty$six) OR (short NEXT/1 form NEXT/1 thirty NEXT/1 six) OR (short NEXT/1 form NEXT/1 thirtysix) OR (short NEXT/1 form NEXT/1 thirty$six)	66,200
#24	(patient* NEAR/2 outcome* NEXT/1 measure*) OR (patient$report* NEXT/1 outcome* NEXT/1 measure*) OR (self$report* NEAR/2 outcome*) OR 'pro'/exp OR pro OR prom OR proms OR hrpro OR hr$pro OR (hr NEXT/1 pro) OR hrprom OR hr$prom OR (hr NEXT/1 prom) OR hrproms OR hr$proms OR (hr NEXT/1 proms)	565,196
#23	patient* NEAR/2 satisfaction	209,198
#22	health NEAR/2 (level* OR qualit* OR state* OR status OR index OR indices OR function* OR outcome* OR profil*)	893,172
#21	qualit* NEAR/2 well NEXT/1 being	3,979
#20	qualit* NEAR/2 (wellbeing OR well$being)	2,260
#19	(qualit* NEAR/2 (life OR living)) OR qol OR hr$qol OR (hr NEXT/1 ql) OR hr$ql OR h$ql OR h$qol	946,732
#18	'european organization for research and treatment of cancer quality of life questionnaire core 30'/exp OR 'european quality of life 5 dimensions questionnaire'/exp OR 'quality of life enjoyment and satisfaction questionnaire'/exp OR 'quality of life index'/exp OR 'short form 36'/exp OR 'whoqol-100'/exp OR 'functional assessment of cancer therapy'/exp OR 'functional assessment of chronic illness therapy'/exp OR 'genital system disease assessment'/mj OR 'expanded prostate cancer index composite'/exp OR 'international prostate symptom score'/mj	93,261
#17	'patient-reported outcome'/mj	21,923
#16	'quality of life'/exp OR 'quality of life assessment'/mj OR 'activity of daily living assessment'/mj OR 'daily life activity'/mj OR 'general health status assessment'/mj OR 'functional status assessment'/mj	771,931
#15	#4 OR #5 OR #6 OR #7 OR #8 OR #9 OR #10 OR #11 OR #12 OR #13 OR #14	947,153
#14	zoladex OR eligard OR fensolvi OR lupron OR lucrin OR viadur OR camcevi OR decapeptyl OR trelstar OR triptodur OR suprefact OR suprecur OR firmagon OR zytiga OR yonsa OR casodex OR cosudex OR calutide OR calumid OR kalumid OR bicadex OR bical OR bicalox OR bicamide OR bicatlon OR bicusan OR binabic OR bypro OR calutol OR ormandyl OR eulexin OR xtandi OR erleada OR nubeqa OR androcur OR androcur$100 OR androstat OR asoteron OR cyprone OR cyproplex OR cyprostat OR cysaxal OR imvel OR siterone OR eliptenor OR cytadren OR orimeten OR belara OR gynorelle OR luteran OR prostal	10,746
#13	goserelin OR leuprolide OR leuprorelin OR leuprolin OR leuprolidine OR triptorelin OR buserelin OR etilamide OR degarelix OR abiraterone OR bicalutamide OR flutamide OR niftolide OR enzalutamide OR apalutamide OR darolutamide OR ketodarolutamide OR darramamide OR cyproterone OR aminoglutethimide OR (chlormadinone NEXT/1 acetate) OR chlormadinone$acetate	70,929
#12	(anti NEXT/1 gonadotropin*) OR antigonadotropin* OR anti$gonadotropin*	193
#11	lute$ni?ing NEAR/1 hormone NEAR/3 (antagonist$ OR agonist$ OR analog*)	2,111

No.	Query	Results
**#10**	(gonadotropin OR gnrh OR lhrh) NEAR/3 (antagonist$ OR agonist$ OR analog*)	26,470
**#9**	hormon* NEAR/3 (drug* OR medication* OR therap* OR treatment* OR agent*)	183,805
**#8**	(androgen* NEAR/3 (inhibit* OR antagonis* OR depriv* OR blocker* OR blockade*)) OR (anti NEXT/1 androgen*) OR antiandrogen* OR anti$androgen* OR (androgen* NEAR/2 receptor* NEAR/2 signal* NEAR/2 inhibit*) OR arsi	53,490
**#7**	'gonadorelin antagonist'/exp	12,935
**#6**	'gonadorelin agonist'/exp	29,730
**#5**	'antineoplastic hormone agonists and antagonists'/exp	730,455
**#4**	'antiandrogen'/exp	8,8132
**#3**	#1 OR #2	366,801
**#2**	prostat* NEAR/3 (cancer* OR carcin* OR adenocarcin* OR neoplas* OR tumo$r* OR anticarcinogen* OR anti$carcionogen* OR malignan* OR oncogen* OR oncolog*)	365,712
**#1**	'prostate tumor'/exp OR 'prostate tumor'	330,017

Table S4. Cochrane Search Strategy (Date Run: 15/04/2025)

ID	Search	Hits
**#1**	MeSH descriptor: [Prostatic Neoplasms] explode all trees	8,980
**#2**	(prostat* NEAR/2 (cancer* or carcin* or adenocarcin* or neoplas* or tumo?r* or anticarcinogen* or anti-carcionogen* or (anti NEXT carcinogen*) or malignan* or oncogen* or oncolog*))	19,501
**#3**	#1 OR #2	19,501
**#4**	MeSH descriptor: [Androgen Antagonists] explode all trees	1,619
**#5**	MeSH descriptor: [Antineoplastic Agents, Hormonal] this term only	2,099
**#6**	MeSH descriptor: [Gonadotropin-Releasing Hormone] explode all trees	3,271
**#7**	((androgen* NEAR/2 (inhibit* or antagonis* or depriv* or blocker* or blockade*)) or (anti NEXT androgen*) or antiandrogen* or anti-androgen* or (androgen* NEAR/1 receptor* NEAR/1 signal* NEAR/1 inhibit*) or arsi)	5,023
**#8**	(hormon* NEAR/2 (drug* or medication* or therap* or treatment* or agent*))	19,072
**#9**	(((gonadotropin or gnrh or ((lute?nizing or lute?nising) NEAR/0 hormone) or lhrh) NEAR/2 (antagonist? or agonist? or analog*)) or (anti NEXT gonadotropin*) or antigonadotropin* or anti-gonadotropin* or gonadorelin)	4,659
**#10**	(goserelin or leuprolide or leuprorelin or leuprolin or leuprolidine or triptorelin or buserelin or etilamide or degarelix or abiraterone or bicalutamide or flutamide or niftolide or enzalutamide or apalutamide or darolutamide or ketodarolutamide or darramamide or cyproterone or aminoglutethimide or (chlormadinone NEXT acetate) or chlormadinone-acetate)	7,519
**#11**	(zoladex or eligard or fensolvi or lupron or lucrin or viadur or camcevi or decapeptyl or trelstar or triptodur or suprefact or suprecur or firmagon or zytiga or yonsa or casodex or cosudex or calutide or calumid or kalumid or bicadex or bical or bicalox or bicamide or bicatlon or bicusan or binabic or bypro or calutol or ormandyl or eulexin or xtandi or erleada or nubeqa or androcur or androcur-100 or androstat or asoteron or cyprone or cyproplex or cyprostat or cysaxal or imvel or siterone or eliptenor or cytadren or orimeten or belara or gynorelle or luteran or prostal)	1,002
**#12**	#4 OR #5 OR #6 OR #7 OR #8 OR #9 OR #10 OR #11	29,859
**#13**	MeSH descriptor: [Quality of Life] explode all trees	44,265
**#14**	MeSH descriptor: [Activities of Daily Living] explode all trees	13,241
**#15**	MeSH descriptor: [Health Status] explode all trees	52,740
**#16**	MeSH descriptor: [Health Care Surveys] this term only	541
**#17**	MeSH descriptor: [Patient Reported Outcome Measures] this term only	1,992
**#18**	((qualit* NEAR/1 (life or living)) or qol or hrqol or (hr NEXT qol) or hr-qol or hrql or (hr NEXT ql) or hr-ql or hql or hqol or (h NEXT qol))	56,928
**#19**	(qualit* NEAR/1 (wellbeing or well-being or (well NEXT being)))	1,346
**#20**	(health NEAR/1 (level* or qualit* or state* or status or index or indices or function* or outcome* or profil*))	43,425
**#21**	(patient* NEAR/1 satisfaction)	40,772
**#22**	((patient* NEAR/1 outcome* NEXT measure*) or (patient-report* NEXT outcome* NEXT measure*) or (self-report* NEAR/1 outcome*) or PRO or PROM or PROMs or hrpro or hr-pro or (hr NEXT pro) or hrprom or hr-prom or (hr NEXT prom) or hrproms or hr-proms or (hr NEXT proms))	35,613

**#23**	(sf36 or sf-36 or (sf NEXT "36") or (short NEXT form NEXT "36") or (shortform NEXT "36") or (short-form NEXT "36") or (sf NEXT thirtysix) or (sf NEXT thirty NEXT six) or (sf NEXT thirty-six) or (shortform NEXT thirtysix) or (shortform NEXT thirty NEXT six) or (shortform NEXT thirty-six) or (short NEXT form NEXT thirty NEXT six) or (short NEXT form NEXT thirtysix) or (short NEXT form NEXT thirty-six))	21,123
**#24**	(sf6 or sf-6 or (sf NEXT "6") or (short NEXT form NEXT "6") or (shortform NEXT "6") or (short-form NEXT "6") or (sf NEXT six) or (shortform NEXT six) or (short NEXT form NEXT six) or (short-form NEXT six))	363
**#25**	(sf12 or sf-12 or (sf NEXT "12") or (short NEXT form NEXT "12") or (shortform NEXT "12") or (short-form NEXT "12") or (sf NEXT twelve) or (shortform NEXT twelve) or (short NEXT form NEXT twelve) or (short-form NEXT twelve))	4,767
**#26**	(sf16 or sf-16 or (sf NEXT "16") or (short NEXT form NEXT "16") or (shortform NEXT "16") or (short-form NEXT "16") or (sf NEXT sixteen) or (sf NEXT six NEXT teen) or (sf NEXT six-teen) or (shortform NEXT sixteen) or (shortform NEXT six NEXT teen) or (shortform NEXT six-teen) or (short NEXT form NEXT six NEXT teen) or (short NEXT form NEXT sixteen) or (short NEXT form NEXT six-teen))	19
**#27**	(sf20 or sf-20 or (sf NEXT "20") or (short NEXT form NEXT "20") or (shortform NEXT "20") or (short-form NEXT "20") or (sf NEXT twenty) or (shortform NEXT twenty) or (short NEXT form NEXT twenty) or (short-form NEXT twenty))	149
**#28**	(euroqol or (euro NEXT qol) or eq5d or (eq NEXT 5d) or eq-5d)	16,712
**#29**	(flic or (functional NEXT living NEXT index NEXT cancer))	68
**#30**	((functional NEXT assessment NEAR/1 chronic NEXT illness NEXT therapy) or facit)	1,880
**#31**	((quality NEAR/1 life NEXT cancer NEXT survivor*) or (quality NEAR/1 life-cancer NEXT survivor*) or qol-cs or (qol NEXT cs))	4
**#32**	((quality NEAR/1 life NEXT index) or qli or qli-cv or (qli NEXT cv))	124
**#33**	((quality NEAR/1 life NEAR/1 adult NEXT cancer NEXT survivor*) or ql-acs or qlacs or (ql NEXT acs))	7
**#34**	((body NEXT image NEAR/1 relationships NEXT scale) or birs)	6
**#35**	((european NEXT organization NEAR/1 research NEAR/1 treatment NEAR/1 cancer NEXT core NEXT "30") or (european NEXT organization NEAR/1 research NEAR/1 treatment NEAR/1 cancer NEXT core-30) or (european NEXT organization NEAR/1 research NEAR/1 treatment NEAR/1 cancer NEXT core30) or (european NEXT organization NEAR/1 research NEAR/1 treatment NEAR/1 cancer NEXT c30) or (eortc NEXT qlq NEXT c NEXT "30") or (eortc NEXT qlq-c-30) or (eortc NEXT qlq-c30) or eortc-qlq-c-30 or eortc-qlq-c30 or (eortc NEXT core NEXT quality NEAR/1 life) or (eortc NEXT core NEXT "30") or (eortc NEXT core30) or (eortc NEXT c30) or (european NEXT organization NEAR/1 research NEAR/1 treatment NEAR/1 cancer NEXT prostate NEXT cancer NEXT "25") or (european NEXT organization NEAR/1 research NEAR/1 treatment NEAR/1 cancer NEXT pr-25) or (european NEXT organization NEAR/1 research NEAR/1 treatment NEAR/1 cancer NEXT pr25) or (european NEXT organization NEAR/1 research NEAR/1 treatment NEAR/1 cancer NEXT pr NEXT "25") or (eortc NEXT qlq NEXT pr NEXT "25") or (eortc NEXT qlq-pr-25) or (eortc NEXT qlq-pr25) or eortc-qlq-pr-25 or eortc-qlq-pr25 or (eortc NEAR/1 pr NEXT "25") or (eortc NEAR/1 pr25))	4,924
**#36**	(fact-g or fact-p or (functional NEXT assessment NEAR/1 cancer NEXT therapy) or (functional NEXT assessment NEAR/1 cancer NEXT therapy NEXT general) or (functional NEXT assessment NEAR/1 cancer NEXT therapy-general) or (functional	1,128

	NEXT assessment NEAR/1 cancer NEXT therapy NEXT prostate) or (functional NEXT assessment NEAR/1 cancer NEXT therapy-prostate))	
**#37**	(escap-cdv or (escap NEXT cdv))	0
**#38**	(epic or (expanded NEXT prostate NEXT cancer NEXT index))	1,542
**#39**	(pc-qol or (pc NEXT qol) or (prostate NEXT cancer NEXT quality NEAR/1 life NEXT instrument))	17
**#40**	(pcsi or (prostate NEXT cancer NEXT symptom NEXT indices))	27
**#41**	(porpus or (patient-oriented NEXT prostate NEXT utility NEXT scale) or (patient NEXT oriented NEXT prostate NEXT utility NEXT scale))	4
**#42**	(ucla-pci or (ucla NEXT pci) or (university NEAR/1 california NEXT los NEXT angeles NEXT prostate NEXT cancer NEXT index) or (university NEAR/1 california NEXT los NEXT angeles-prostate NEXT cancer NEXT index))	27
**#43**	(WHOQOL-100 or WHO-QOL-100 or WHO-QOL100 or (WHOQOL NEXT "100") or (WHO NEXT QOL NEXT "100") or (WHO-QOL NEXT "100") or (WHO NEXT QOL100) or (WHO NEXT QOL-100) or (world NEXT health NEXT organi?ation NEXT quality NEAR/1 life NEXT "100") or (world NEXT health NEXT organi?ation-quality NEAR/1 life NEXT "100") or (world NEXT health NEXT organi?ation NEXT quality NEAR/1 life-100) or (world NEXT health NEXT organi?ation-quality NEAR/1 life-100) or WHOQOL-BREF or WHO-QOL-BREF or (WHOQOL NEXT BREF) or (WHO NEXT QOL NEXT BREF) or (WHO-QOL NEXT BREF) or (WHO NEXT QOL-BREF) or (world NEXT health NEXT organi?ation NEXT quality NEAR/1 life NEXT BREF) or (world NEXT health NEXT organi?ation-quality NEAR/1 life NEXT BREF) or (world NEXT health NEXT organi?ation NEXT quality NEAR/1 life-BREF) or (world NEXT health NEXT organi?ation-quality NEAR/1 life-BREF))	1,820
**#44**	IPSS or (international NEXT prostate NEXT symptom NEXT score)	3,966
**#45**	#13 #14 OR #15 #16 OR #17 #18 OR #19 OR #20 OR #21 OR #22 OR #23 OR #24 OR #25 OR #26 OR #27 OR #28 OR #29 OR #30 OR #31 OR #32 OR #33 OR #34 OR #35 OR #36 OR #37 OR #38 OR #39 OR #40 OR #41 OR #42 #43 OR #44	154,754
**#46**	#3 AND #12 AND #45	894

Table S5. CINAHL Search Strategy

#	Query	Limiters/Expanders	Results
**S44**	S3 AND S12 AND S43	Expanders - Apply equivalent subjects<br>Narrow by Language: - english<br>Search modes - Find all my search terms	1,128
**S43**	S13 OR S14 OR S15 OR S16 OR S17 OR S18 OR S19 OR S20 OR S21 OR S22 OR S23 OR S24 OR S25 OR S26 OR S27 OR S28 OR S29 OR S30 OR S31 OR S32 OR S33 OR S34 OR S35 OR S36 OR S37 OR S38 OR S39 OR S40 OR S41 OR S42	Expanders - Apply equivalent subjects<br>Search modes - Find all my search terms	780,687
**S42**	IPSS OR (international W0 prostate W0 symptom W0 score)	Expanders - Apply equivalent subjects<br>Search modes - Find all my search terms	1,218
**S41**	(WHOQOL-100 or WHO-QOL-100 or WHO-QOL100 or (WHOQOL W0 "100") or (WHO W0 QOL W0 "100") or (WHO-QOL W0 "100") or (WHO W0 QOL100) or (WHO W0 QOL-100) or (world W0 health W0 organi#ation W0 quality N1 life W0 "100") or (world W0 health W0 organi#ation-quality N1 life W0 "100") or (world W0 health W0 organi#ation W0 quality N1 life-100) or (world W0 health W0 organi#ation-quality N1 life-100) or WHOQOL-BREF or WHO-QOL-BREF or (WHOQOL W0 BREF) or (WHO W0 QOL W0 BREF) or (WHO-QOL W0 BREF) or (WHO W0 QOL-BREF) or (world W0 health W0 organi#ation W0 quality N1 life W0 BREF) or (world W0 health W0 organi#ation-quality N1 life W0 BREF) or (world W0 health W0 organi#ation W0 quality N1 life-BREF) or (world W0 health W0 organi#ation-quality N1 life-BREF))	Expanders - Apply equivalent subjects<br>Search modes - Find all my search terms	1,925
**S40**	(ucla-pci or (ucla W0 pci) or (university N1 california W0 los W0 angeles W0 prostate W0 cancer W0 index) or (university N1 california W0 los W0 angeles-prostate W0 cancer W0 index))	Expanders - Apply equivalent subjects<br>Search modes - Find all my search terms	38
**S39**	(porpus or (patient-oriented W0 prostate W0 utility W0 scale) or (patient W0 oriented W0 prostate W0 utility W0 scale))	Expanders - Apply equivalent subjects<br>Search modes - Find all my search terms	12
**S38**	(pcsi OR (prostate W0 cancer W0 symptom W0 indices))	Expanders - Apply equivalent subjects<br>Search modes - Find all my search terms	62
**S37**	(pc-qol or (pc W0 qol) or (prostate W0 cancer W0 quality N1 life W0 instrument))	Expanders - Apply equivalent subjects<br>Search modes - Find all my search terms	8
**S36**	(epic OR (expanded W0 prostate W0 cancer W0 index))	Expanders - Apply equivalent subjects<br>Search modes - Find all my search terms	2,117
**S35**	(escap-cdv or (escap W0 cdv))	Expanders - Apply equivalent subjects<br>Search modes - Find all my search terms	0
**S34**	(fact-g or fact-p or (functional W0 assessment N1 cancer W0 therapy) or (functional W0 assessment	Expanders - Apply equivalent subjects<br>Search modes - Find all my search terms	1,575

	N1 cancer W0 therapy W0 general) or (functional W0 assessment N1 cancer W0 therapy-general) or (functional W0 assessment N1 cancer W0 therapy W0 prostate) or (functional W0 assessment N1 cancer W0 therapy-prostate))		
**S33**	((european W0 organization N1 research N1 treatment N1 cancer W0 core W0 "30") or (european W0 organization N1 research N1 treatment N1 cancer W0 core-30) or (european W0 organization N1 research N1 treatment N1 cancer W0 core30) or (european W0 organization N1 research N1 treatment N1 cancer W0 c30) or (eortc W0 qlq W0 c W0 "30") or (eortc W0 qlq-c-30) or (eortc W0 qlq-c30) or eortc-qlq-c-30 or eortc-qlq-c30 or (eortc W0 core W0 quality N1 life) or (eortc W0 core W0 "30") or (eortc W0 core30) or (eortc W0 c30) or (european W0 organization N1 research N1 treatment N1 cancer W0 prostate W0 cancer W0 "25") or (european W0 organization N1 research N1 treatment N1 cancer W0 pr-25) or (european W0 organization N1 research N1 treatment N1 cancer W0 pr25) or (european W0 organization N1 research N1 treatment N1 cancer W0 pr W0 "25") or (eortc W0 qlq W0 pr W0 "25") or (eortc W0 qlq-pr-25) or (eortc W0 qlq-pr25) or eortc-qlq-pr-25 or eortc-qlq-pr25 or (eortc N1 pr W0 "25") or (eortc N1 pr25))	Expanders - Apply equivalent subjects Search modes - Find all my search terms	1,643
**S32**	((body W0 image N1 relationships W0 scale) or birs)	Expanders - Apply equivalent subjects Search modes - Find all my search terms	14,385
**S31**	((quality N1 life N1 adult W0 cancer W0 survivor*) or ql-acs or qlacs or (ql W0 acs))	Expanders - Apply equivalent subjects Search modes - Find all my search terms	35
**S30**	((quality N1 life W0 index) or qli or qli-cv or (qli W0 cv))	Expanders - Apply equivalent subjects Search modes - Find all my search terms	39,821
**S29**	((quality N1 life W0 cancer W0 survivor*) or (quality N1 life-cancer W0 survivor*) or qol-cs or (qol W0 cs))	Expanders - Apply equivalent subjects Search modes - Find all my search terms	30
**S28**	((functional W0 assessment N1 chronic W0 illness W0 therapy) or facit)	Expanders - Apply equivalent subjects Search modes - Find all my search terms	786
**S27**	(flic or (functional W0 living W0 index W0 cancer))	Expanders - Apply equivalent subjects Search modes - Find all my search terms	81
**S26**	(euroqol or (euro W0 qol) or eq5d or (eq W0 5d) or eq-5d)	Expanders - Apply equivalent subjects Search modes - Find all my search terms	5,818
**S25**	(sf20 or sf-20 or (sf W0 "20") or (short W0 form W0 "20") or (shortform W0 "20") or (short-form W0 "20") or (sf W0 twenty) or (shortform W0 twenty) or (short W0 form W0 twenty) or (short-form W0 twenty))	Expanders - Apply equivalent subjects Search modes - Find all my search terms	73

**S24**	(sf16 or sf-16 or (sf W0 "16") or (short W0 form W0 "16") or (shortform W0 "16") or (short-form W0 "16") or (sf W0 sixteen) or (sf W0 six W0 teen) or (sf W0 six-teen) or (shortform W0 sixteen) or (shortform W0 six W0 teen) or (shortform W0 six-teen) or (short W0 form W0 six W0 teen) or (short W0 form W0 sixteen) or (short W0 form W0 six-teen))	Expanders - Apply equivalent subjects Search modes - Find all my search terms	5
**S23**	(sf12 or sf-12 or (sf W0 "12") or (short W0 form W0 "12") or (shortform W0 "12") or (short-form W0 "12") or (sf W0 twelve) or (shortform W0 twelve) or (short W0 form W0 twelve) or (short-form W0 twelve))	Expanders - Apply equivalent subjects Search modes - Find all my search terms	3,324
**S22**	(sf6 or sf-6 or (sf W0 "6") or (short W0 form W0 "6") or (shortform W0 "6") or (short-form W0 "6") or (sf W0 six) or (shortform W0 six) or (short W0 form W0 six) or (short-form W0 six))	Expanders - Apply equivalent subjects Search modes - Find all my search terms	158
**S21**	(sf36 or sf-36 or (sf W0 "36") or (short W0 form W0 "36") or (shortform W0 "36") or (short-form W0 "36") or (sf W0 thirtysix) or (sf W0 thirty W0 six) or (sf W0 thirty-six) or (shortform W0 thirtysix) or (shortform W0 thirty W0 six) or (shortform W0 thirty-six) or (short W0 form W0 thirty W0 six) or (short W0 form W0 thirtysix) or (short W0 form W0 thirty-six))	Expanders - Apply equivalent subjects Search modes - Find all my search terms	19,067
**S20**	((patient* N1 outcome* W0 measure*) or (patient-report* W0 outcome* W0 measure*) or (self-report* N1 outcome*) or PRO or PROM or PROMs or hrpro or hr-pro or (hr W0 pro) or hrprom or hr-prom or (hr W0 prom) or hrproms or hr-proms or (hr W0 proms))	Expanders - Apply equivalent subjects Search modes - Find all my search terms	45,821
**S19**	(patient* N1 satisfaction)	Expanders - Apply equivalent subjects Search modes - Find all my search terms	72,248
**S18**	(health N1 (level* or qualit* or state* or status or index or indices or function* or outcome* or profil*))	Expanders - Apply equivalent subjects Search modes - Find all my search terms	337,190
**S17**	(qualit* N1 (wellbeing or well-being or (well W0 being)))	Expanders - Apply equivalent subjects Search modes - Find all my search terms	1,786
**S16**	((qualit* N1 (life or living)) or qol or hrqol or (hr W0 qol) or hr-qol or hrql or (hr W0 ql) or hr-ql or hql or hqol or (h W0 qol))	Expanders - Apply equivalent subjects Search modes - Find all my search terms	243,245
**S15**	(MH "Patient-Reported Outcomes") OR (MH "Self Report")	Expanders - Apply equivalent subjects Search modes - Find all my search terms	89,141
**S14**	(MH "Health Status+")	Expanders - Apply equivalent subjects Search modes - Find all my search terms	140,543
**S13**	(MH "Quality of Life+") OR (MH "Health and Life Quality (Iowa NOC)+") OR (MH "Ferrans and Powers Quality of Life Index") OR (MH	Expanders - Apply equivalent subjects Search modes - Find all my search terms	222,294

	"Psychological Well-Being") OR (MH "Psychological Well-Being (Iowa NOC)+")		
**S12**	S4 OR S5 OR S6 OR S7 OR S8 OR S9 OR S10 OR S11	Expanders - Apply equivalent subjects<br>Search modes - Find all my search terms	47,978
**S11**	(zoladex or eligard or fensolvi or lupron or lucrin or viadur or camcevi or decapeptyl or trelstar or triptodur or suprefact or suprecur or firmagon or zytiga or yonsa or casodex or cosudex or calutide or calumid or kalumid or bicadex or bical or bicalox or bicamide or bicatlon or bicusan or binabic or bypro or calutol or ormandyl or eulexin or xtandi or erleada or nubeqa or androcur or androcur-100 or androstat or asoteron or cyprone or cyproplex or cyprostat or cysaxal or imvel or siterone or eliptenor or cytadren or orimeten or belara or gynorelle or luteran or prostal)	Expanders - Apply equivalent subjects<br>Search modes - Find all my search terms	790
**S10**	(goserelin or leuprolide or leuprorelin or leuprolin or leuprolidine or triptorelin or buserelin or etilamide or degarelix or abiraterone or bicalutamide or flutamide or niftolide or enzalutamide or apalutamide or darolutamide or ketodarolutamide or darramamide or cyproterone or aminoglutethimide or (chlormadinone W0 acetate) or chlormadinone-acetate)	Expanders - Apply equivalent subjects<br>Search modes - Find all my search terms	3,284
**S9**	(((gonadotropin or gnrh or ((lute#nizing or lute#nising) N0 hormone) or lhrh) N2 (antagonist# or agonist# or analog*)) or (anti W0 gonadotropin*) or antigonadotropin* or anti-gonadotropin* or gonadorelin)	Expanders - Apply equivalent subjects<br>Search modes - Find all my search terms	2,935
**S8**	(hormon* N2 (drug* or medication* or therap* or treatment* or agent*))	Expanders - Apply equivalent subjects<br>Search modes - Find all my search terms	38,812
**S7**	((androgen* N2 (inhibit* or antagonis* or depriv* or blocker* or blockade*)) or (anti W0 androgen*) or antiandrogen* or anti-androgen* or (androgen* N1 receptor* N1 signal* N1 inhibit*) or arsi)	Expanders - Apply equivalent subjects<br>Search modes - Find all my search terms	5,492
**S6**	(MH "Gonadotropin-Releasing Hormone+")	Expanders - Apply equivalent subjects<br>Search modes - Find all my search terms	2,713
**S5**	(MH "Antineoplastic Agents, Hormonal+")	Expanders - Apply equivalent subjects<br>Search modes - Find all my search terms	5,716
**S4**	(MH "Androgen Antagonists+")	Expanders - Apply equivalent subjects<br>Search modes - Find all my search terms	3,293
**S3**	S1 OR S2	Expanders - Apply equivalent subjects<br>Search modes - Find all my search terms	49,440
**S2**	(prostat* N2 (cancer* or carcin* or adenocarcin* or neoplas* or tumo#r* or anticarcinogen* or anti-	Expanders - Apply equivalent subjects<br>Search modes - Find all my search terms	49,440

	carcionogen* or (anti W0 carcinogen*) or malignan* or oncogen* or oncolog*))		
**S1**	(MH "Prostatic Neoplasms+")	Expanders - Apply equivalent subjects<br>Search modes - Find all my search terms	35,497

Supplementary File S1. Full-text screening form

First Author: **Year:** **Screener Initials:**

1. Is the study population prostate cancer survivors?

☐ No → Exclude

☐ Yes → Go to the next question

2. Is the exposure/intervention hormonal treatment?

☐ No → Exclude

☐ Yes → Go to the next question

3. Is the surgery, radiation therapy, cyberknife therapy, cryotherapy, chemotherapy, targeted therapy, or comparator active surveillance/watchful waiting/ no treatment?

☐ No → Exclude

☐ Yes → Go to the next question

4. Are the outcomes general HRQOL and/or prostate-cancer specific QOL/ symptom burden 5 years post-diagnosis?

☐ No → Exclude

☐ Yes → Include

Table S6. Reasons for exclusion

Author	Year	Reason for exclusion
Cappuccio	2025	Follow-up less than 5 years
Devlies	2025	Follow-up less than 5 years
Dickstein	2025	Follow-up less than 5 years
Freedland	2025	Not comparison of interest
Kinnaird	2025	No information on date since diagnosis
Nabid	2025	Not comparison of interest
O'Sullivan	2025	Not outcome of interest
Pembroke	2025	Not outcome of interest
Basu	2024	Conference Abstract
Bultijnck	2024	Not comparison of interest
Calleja-Escudero	2024	Follow-up less than 5 years
Dissing	2024	Follow-up less than 5 years
Hsueh	2024	Follow-up less than 5 years
Hsueh	2024	Follow-up less than 5 years
Hu	2024	Not outcome of interest
Lim	2024	Follow-up less than 5 years
Mittal	2024	Follow-up less than 5 years
Rajeev-Kumar	2024	Not outcome of interest
Tiruye	2024	Follow-up less than 5 years
Karaihira	2023	Not population of interest
Yang	2023	Follow-up less than 5 years
Payne	2022	Follow-up less than 5 years
Sauer	2022	Not population of interest
Thiery-Vuillemin	2022	Follow-up less than 5 years
van Rensburg	2022	Not comparison of interest and follow-up less than 5 years
Adam	2021	Not outcome of interest
Roy	2021	Conference Abstract
Schubbe	2021	Not comparison of interest
Baden	2020	Not outcome of interest
Fizazi	2020	Follow-up less than 5 years
Selvi	2020	Follow-up less than 5 years
Uemura	2020	Follow-up less than 5 years
Alsinnawi	2019	Not comparison of interest
Dearden	2019	Not comparator of interest
Downing	2019	Follow-up less than 5 years
Huang	2019	Not exposure/intervention of interest and follow-up less than 5 years
Kim	2019	Follow-up less than 5 years
Kokkonen	2019	Not population of interest
Maguire	2019	Not outcome of interest
Oraa-Tabernero	2019	Not English
Tombal	2019	Not comparator of interest and follow-up less than 5 years
Vargas	2019	Not comparison of interest and follow-up less than 5 years
Wilding	2019	Not population of interest
Gagliano-Juca	2018	Follow-up less than 5 years

Ueno	2018	Follow-up less than 5 years
Washino	2018	Not comparison of interest and follow-up less than 5 years
Beer	2017	Not comparison of interest
Buzaglo	2017	Conference Abstract
Hammerer	2017	Conference Abstract
Kim	2017	Not comparison of interest
Tripp	2017	Not exposure/intervention of interest and follow-up less than 5 years
Zajdlewicz	2017	Not comparison of interest
Fossa	2016	Non-validated questionnaire
Gavin	2016	Not comparison of interest
Klaff	2016	Not comparator of interest
Manokumar	2016	Follow-up less than 5 years
Niraula	2016	Not comparison of interest and follow-up less than 5 years
Schweizer	2016	Not comparison of interest
Shahrokni	2016	Review
Watson	2016	Not population of interest
Cella	2015	Follow-up less than 5 years
Gavin	2015	Duplicate sample
Hampson	2015	Not comparison of interest and follow-up less than 5 years
Lee	2015	Follow-up less than 5 years
O'Leary	2015	Duplicate sample
Bennedict	2014	Not exposure/intervention of interest
Hammerer	2014	Conference Abstract
Basch	2013	Follow-up less than 5 years
Chipperfield	2013	Follow-up less than 5 years
Wiechno	2013	Follow-up less than 5 years
Ng	2012	Not comparison of interest and follow-up less than 5 years
Sevilla	2012	Follow-up less than 5 years
Storey	2012	Not comparison of interest and follow-up less than 5 years
Kasperzyk	2011	Not comparison of interest
Sadetsky	2011	Follow-up less than 5 years
Wadhwa	2011	Not comparison of interest and follow-up less than 5 years
Zhou	2011	Not population of interest
Gore	2010	Not exposure/intervention of interest and follow-up less than 5 years
Huang	2010	Follow-up less than 5 years
Galvao	2009	Not exposure/intervention of interest
Harrington	2009	Not population of interest
Sartor	2009	Conference Abstract
Jagodic	2008	Not comparator of interest and follow-up less than 5 years
Nishimura	2008	Follow-up less than 5 years
Sanda	2008	Not exposure/intervention of interest
Shimizu	2008	Not comparison of interest
Soyupeck	2008	Follow-up less than 5 years
Sugimoto	2008	Not population of interest
van Andel	2008	Not comparison of interest and follow-up less than 5 years
White	2008	Follow-up less than 5 years
Wu	2008	Not comparison of interest and follow-up less than 5 years

Kakehi	2007	Follow-up less than 5 years
Mols	2007	Not population of interest
Namiki	2007	Not population of interest
Stephens	2007	Not comparison of interest and follow-up less than 5 years
Joly	2006	Follow-up less than 5 years
Gray	2005	Not comparison of interest
Krupski	2005	Not population of interest
Nishimura	2005	Follow-up less than 5 years
Nishiyama	2004	Not comparison of interest
Oefelein	2003	Not comparison of interest and follow-up less than 5 years
van Andel	2003	Follow-up less than 5 years
Basaria	2002	Not population of interest
Lubeck	2001	Follow-up less than 5 years
Herr	2000	Not exposure/intervention of interest and follow-up less than 5 years
Iversen	2000	Wrong study design/Pooled analysis
Smith	2000	Follow-up less than 5 years
Rosendahl	1999	Not outcome of interest
Litwin	1998	Follow-up less than 5 years
Tyrrell	1998	Not comparator of interest and follow-up less than 5 years
Albertsen	1997	Not comparison of interest
Fossa	1997	Non-validated questionnaire
Kornblith	1994	Follow-up less than 5 years
Herr	1993	Follow-up less than 5 years

Table S7. Risk of bias and quality assessment of included cross-sectional studies using the Joanna Briggs Institute (JBI) critical appraisal tool [2]

JBI Critical Appraisal Checklist for cross-sectional studies	Chowdhury [3]	Jackson [4]	Adam [5]	Kerleau [6]	Carlsson [7]	Drummond [8]	Mols [9]	Voerman [10]	Fowler [11]
**1. Were the criteria for inclusion in the sample clearly defined?**	Yes	Yes	Yes	Yes	Yes	Yes	Yes	Yes	Yes
**2. Were the study subjects and the setting described in detail?**	No	Yes	Yes	Yes	Yes	Yes	Yes	No	Yes
**3. Was the exposure measured in a valid and reliable way? [1]**	No	Yes	No	Yes	Yes	No	Yes	No	No
**4. Were objective, standard criteria used for measurement of the condition?**	Yes	Yes	Yes	Yes	Yes	Yes	Yes	Yes	Yes
**5. Were confounding factors identified? [2]**	No	No	Yes	Yes	Yes	Yes	Yes	No	No
**6. Were strategies to deal with confounding factors stated?**	No	Yes	Yes	Yes	Yes	Yes	Yes	Yes	Yes
**7. Were the outcomes measured in a valid and reliable way?**	Yes	Yes	Yes	Yes	Yes	Yes	Yes	Yes	No
**8. Was appropriate statistical analysis used?**	No	Yes	Yes	Yes	Yes	Yes	Yes	Yes	Yes
**Score**	37.5%	87.5%	87.5%	100%	100%	87.5%	100%	62.5%	62.5%
**Risk of bias [3]**	high	low	low	low	low	low	low	moderate	moderate

[1] 'Yes' if medical records, 'No' if self-reported

[2] 'Yes' if age and stage are included, or stage is pre-defined at recruitment, 'No' if no confounding factors identified, or stage not included

[3] Low if >80%, moderate if 51-80%, and high if ≤50%

Table S8. Risk of bias and quality assessment of included cohort studies using the Joanna Briggs Institute (JBI) critical appraisal tool [2]

JBI Critical Appraisal Checklist for cohort studies	Al Hussein [12]	Gaither [13]	Mazariego [14]	Hammerer [15]	Punnen [16]
**1. Were the two groups similar and recruited from the same population?**	Yes	Yes	Yes	Yes	Yes
**2. Were the exposures measured similarly to assign people to both exposed and unexposed groups?**	Yes	Yes	Yes	Yes	Yes
**3. Was the exposure measured in a valid and reliable way? [1]**	Yes	Yes	Yes	Yes	Yes
**4. Were confounding factors identified? [2]**	Yes	Yes	Yes	No	Yes
**5. Were strategies to deal with confounding factors stated?**	Yes	Yes	Yes	No	Yes
**6. Were the groups/participants free of the outcome at the start of the study (or at the moment of exposure)? [3]**	NA	NA	NA	NA	NA
**7. Were the outcomes measured in a valid and reliable way?**	Yes	Yes	Yes	Yes	Yes
**8. Was the follow up time reported and sufficient to be long enough for outcomes to occur?**	Yes	Yes	Yes	Yes	Yes
**9. Was follow up complete, and if not, were the reasons to loss to follow up described and explored?**	Yes	No	Yes	No	Yes
**10. Were strategies to address incomplete follow up utilized?**	Yes	No	Yes	No	Yes
**11. Was appropriate statistical analysis used?**	Yes	Yes	Yes	No	Yes
**Score**	100%	80%	100%	50%	100%
**Risk of bias [4]**	low	moderate	low	high	low

[1] 'Yes' if medical records, 'No' if self-reported
[2] 'Yes' if age and stage are included, or stage is pre-defined at recruitment, 'No' if no confounding factors identified, or stage not included
[3] Not applicable because the outcome is quality of life at follow-up
[4] Low if >80%, moderate if 51-80%, and high if ≤50

Table S9. Characteristics of the included studies

Author<br>Country<br>Year	Study design	Sampling Method	Stage and/or Grade	Hormone treatment(s)	Comparator treatment(s)	Sample size	Age<br>mean (SD) or median [IQR] (years)	Questionnaire(s)	Covariates	Outcome(s)	Timepoint(s)<br>Or time since diagnosis
Al Hussein [12]<br>USA<br>2024	Cohort	Men ≤ 80 years from 5 population-based registries within 6 months of PC diagnosis in 2011-2012	T2N0M0<br>Grade: 3-5	EBRT + ADT	RP	568	EBRT+ADT: 64 [59-68] at diagnosis<br>RP: 71 [66-74] at diagnosis	EPIC-26<br>SF-36<br>SF-12	Age at diagnosis, race, comorbidity score, PC risk group, SEER site, treatment modality, time since treatment, receipt of ADT within 1 year from treatment, baseline EPIC-26 domain scores, and baseline physical functioning, social support, depression, and participatory decision-making	1. PC-specific QOL<br>2. General HRQOL	Baseline, 6 months, 1, 3, 5, and 10 years after treatment
Chowdhury [3]<br>UK<br>2024	Cross-sectional	Men with PC 0 – 10+ years post-diagnosis, recruited using a convenience approach by 3 PC charities and members of PC support groups	1-4	Ever-HT (ADT or novel AA)	HT-naive	286	67 (7.81) at survey	FACT-G<br>FACT-P<br>DASS-21<br>MSE scale<br>CompACT processes measures	Age	1. General HRQOL<br>2. Prostate cancer symptoms<br>3. Distress<br>4. Masculine self-esteem<br>5. Psychological flexibility	0-2 years, 2-4.9, 5-9.9, and 10+ years since diagnosis (mean: 4.9 years)
Gaither [13]<br>USA<br>2022	Prospective cohort	Men with biopsy-proven PC receiving care as part of a nationwide disease-specific healthcare program enrolled from 2001 to January 2020	Metastatic	Medical castration	Bilateral orchiectomy	301	61.3 [56–65] at study enrollment	SF-12<br>UCLA PCI-SF	Age at diagnosis, education level, relationship status, pre-treatment PSA, primary treatment received	1. General HRQOL<br>2. PC-specific QOL	At study enrolment then every 6 months up to 5 years
Jackson [4]<br>Jamaica<br>2022	Case-control/ cross-sectional	40- to 80- year old men with newly diagnosed histologically confirmed PC between March 2005 and July 2007, recruited from urology clinics at two main tertiary hospitals and from private practitioners	All grades	HT (ADT ± oral AA, or orchiectomy) ± EBRT	No-HT (RP and RT)<br><br>Controls (without PC)	143	All PC: 64.3 (7.9) at diagnosis<br>Control: 58.8 (9.6) at diagnosis	EORTC QLQ-C30	Current age, marital status, number of comorbidities, prostate cancer survivor (PC-free/PC survivor), ADT (PC-free/ADT) or non-ADT (PC-free/non-ADT)	General HRQOL	5 to >10 years post-diagnosis

Author<br>Country<br>Year	Study design	Sampling Method	Stage<br>and/or Grade	Hormone treatment(s)	Comparator treatment(s)	Sample size	Age<br>mean (SD) or median [IQR] (years)	Questionnaire(s)	Covariates	Outcome(s)	Timepoint(s)<br>Or time since diagnosis
Mazariego [14]<br>Australia<br>2020	Prospective cohort	Histopathologic confirmed PC <70 years, diagnosed between 2000 and 2002 identified through a cancer registry and controls selected from electoral rolls	T1-T2	ADT ± EBRT	WW/AS, NSRP, Non-NSRP, RP type unknown, EBRT/HDR, LDR, orchidectomy, unknown primary treatment, and age-matched controls	2 137	75.9 at 15 years post-diagnosis (range: 50-85)	UCLA PCI<br>EPIC-26<br>SF-12	Baseline age, marital status, having private health insurance, region of residence, income, education, country of birth, comorbidity score, and domain-specific baseline QOL score	1. PC-specific QOL<br>2. General HRQOL	Baseline, 1, 2, 3, 5, 10, and 15 years post-diagnosis (median: 15.2 years)
Adam [5]<br>Germany<br>2019	Cross-sectional	PC survivors diagnosed between 1994 and 2004, identified from multiregional cancer registries	T1-T2N0M0	ADT (± RP/RT)	RP, RT, RP & RT, NTX	911	65.1 (5.5) at diagnosis	EORTC QLQ-C30<br>EORTC QLQ-PR25	Education, number of comorbidities at diagnosis, years since diagnosis and age at diagnosis	1. General HRQOL<br>2. PC-specific QOL/symptom burden	5-15 years post-diagnosis
Hammerer [15]<br>Germany<br>2018	Retrospective and prospective cohort	PC patients and cancer- and ADT-free age-matched controls from 30 office-based urologists	Advanced and hormone-sensitive	ADT (leuprorelin) ± AA or RT or other TX	No ADT	652	ADT: 79.6 (6.3) at follow-up visit<br>No ADT: 80.5 (3.1) at follow-up visit	EORTC QLQ-C30<br>EORTC QLQ-PR25	-	1. General HRQOL<br>2. PC-specific QOL	5-19.8 years after ADT treatment (mean treatment duration: 8.6 years)
Kerleau [6]<br>France<br>2016	Case-control/<br>cross-sectional	Long-term PC patients, survivors over 40 years initially enrolled in a large cohort from in 2001 from 11 cancer registries and controls selected from electoral rolls	T1-T2	ADT at survey	No ADT at survey	574	PC: 75.8 [61-91]<br>Controls: 76.5 [59-91]	EORTC QLQ-C30<br>EPIC<br>HADS	Age, Gleason score, regular medication	1. General HRQOL<br>2. PC-specific QOL<br>3. Anxiety and depression	10 years post-diagnosis
Carlsson [7]<br>Sweden<br>2016	Case-Control/<br>cross-sectional	Men diagnosed with PC between 1997 and 2002, recruited from a national PC registry and controls from the general population	T1-T2N0M0<br>Low and intermediate risk	ADT, RP+ADT, RT+ADT, RP+RT+ADT	Surveillance, RP, RT, RP+RT, age-matched controls	6 944	PC patients: 63 [59-67] at diagnosis<br>Controls: 74 [70-78]	Questionnaire based on the Prostate-Cancer Symptom Scale self-assessment, formerly the Questionnaire Umea Fransson Widmark 1994	Age	PC-specific symptom burden	11-13 years post-diagnosis

Author<br>Country<br>Year	Study design	Sampling Method	Stage<br>and/or Grade	Hormone treatment(s)	Comparator treatment(s)	Sample size	Age<br>mean (SD) or median [IQR] (years)	Questionnaire(s)	Covariates	Outcome(s)	Timepoint(s)<br>Or time since diagnosis
Punnen [16]<br>USA<br>2015	Prospective cohort	Biopsy-proven PC patients from 45 predominantly community-based urology practices, diagnosed from 1995 to 2011	T1-T3	Primary ADT	NSRP, non-NSRP, BT, EBRT, WW/AS	3 294	Primary ADT: 73.6 (8.1) at diagnosis<br>NSRP: 60.0 (6.8) at diagnosis<br>non-NSRP: 62.9 (6.6) at diagnosis<br>BT: 68.3 (7.2) at diagnosis<br>EBRT: 71.3 (6.2) at diagnosis<br>WW/AS: 72.5 (7.9) at diagnosis	SF-36<br>UCLA PCI	Age, comorbidities, receipt of neoadjuvant, adjuvant, or salvage treatment	1. General HRQOL<br>2. PC-specific QOL	Baseline, 2, 5 and 10-year post-treatment
Drummond [8]<br>Ireland<br>2015	Cross-sectional	Men with invasive PC, diagnosed between 1995 and 2010, recruited from population-based cancer registries	I – IV<br>All grades	ADT alone<br>EBRT with ADT	RP, EBRT without ADT, BT, observation, chemotherapy, unknown treatment	3 348	Not reported	EORTC QLQ-C30<br>EORTC QLQ-PR25	Age at diagnosis, time since diagnosis, jurisdiction, comorbidity at diagnosis, mode of presentation, stage, Gleason grade at diagnosis, stage, recurrence, marital status, education level, employment status at questionnaire completion	1. General HRQOL<br>2. PC-specific QOL	2-18 years post-diagnosis (<5 and ≥5 years)
Mols [9]<br>The Netherlands<br>2006	Cross-sectional	Men diagnosed with PC between 1994 and 1998, selected from a population-based cancer registry	I-IV	Primary HT	RP, RT, WW	572	Not reported	SF-36<br>QoL-CS scale	Stage, age at diagnosis, tumor grade, years since diagnosis, education, marital status, and comorbidity	1. General HRQOL<br>2. Generic HRQL survival issues	5-10 years post-diagnosis
Voerman [10]<br>The Netherlands<br>2006	Cross-sectional	Men with PC recruited from 5 hospitals	Local and metastatic	HT	RP, RT, RP & RT, WW	238	67.9 (8.2)	EORTC QLQ-C30<br>EORTC QLQ-PR25	Age	1. General HRQOL<br>2. PC-specific QOL	44.3 (31.3) with a range of 1-139 months since diagnosis

Author Country Year	Study design	Sampling Method	Stage and/or Grade	Hormone treatment(s)	Comparator treatment(s)	Sample size	Age mean (SD) or median [IQR] (years)	Questionnaire(s)	Covariates	Outcome(s)	Timepoint(s) Or time since diagnosis
Fowler [11] USA 2002	Cross-sectional with a retrospective cohort selection	Men with PC who underwent RP between 1991 and 1992, identified from a national database	Not reported	ADT group (medical and surgical castration), currently or since RP	Never-ADT group	1 393	Not reported	Questions were based on previous Patient Outcome Research Team (PORT) for Prostatic Diseases treatment outcome studies of Medicare beneficiaries who had undergone surgery or received radiation for prostate carcinoma, the Health Insurance Study conducted by the RAND corporation, and SF-12	Age	1. Seven HRQOL measures (impact of cancer and treatment, concern regarding body image, mental health, general health, activity, worries about cancer and dying, and energy) 2. Treatment side effects	7-8 years post RP

AA: Antiandrogens; ADT: Androgen Deprivation Therapy; AS: Active Surveillance; BT: Brachytherapy; EBRT: External Beam Radiation Therapy; CompACT: Comprehensive Assessment of Acceptance and Commitment Therapy; DASS-21: Depression, Anxiety, and Stress Scale - 21 items; EORTC QLQ-C30: European Organization for Research and Treatment of Cancer Quality of Life questionnaire; EORTC QLQ-PR25: European Organization for Research and Treatment of Cancer Quality of Life Questionnaire-Prostate 25; EPIC: Expanded Prostate Cancer Index Composite; FACT-G: Functional Assessment of Cancer Therapy - General; FACT-P: Functional Assessment of Cancer Therapy - Prostate; HADS: Hospital Anxiety and Depression Scale; HT: Hormone Treatment; HRQOL: Health-Related Quality of Life; IQR: Interquartile Range; MSE: Masculine Self Esteem; NSRP: Nerve-Sparing Radical Prostatectomy; NTX: No Treatment Reported; PC: Prostate Cancer; PCSS: Prostate Cancer Symptom Scale; QOL: Quality of Life; QoL-CS: Quality of Life Cancer Survivors; RP: Radical Prostatectomy; RT: Radiation Therapy; SD: Standard Deviation; SF-12: The 12-Item Short Form Health Survey; SF-36: The 36-Item Short Form Health Survey; TX: Treatment; UCLA PCI: University of California Los Angeles Prostate Cancer Index; WW: Watchful Waiting

References

1. Tricco, A.C.; Lillie, E.; Zarin, W.; O'Brien, K.K.; Colquhoun, H.; Levac, D.; Moher, D.; Peters, M.D.J.; Horsley, T.; Weeks, L.; et al. PRISMA Extension for Scoping Reviews (PRISMA-ScR): Checklist and Explanation. *Ann Intern Med* **2018**, *169*, 467–473, doi:https://doi.org/10.7326/M18-0850.
2. Moola S, M.Z., Tufanaru C, Aromataris E, Sears K, Sfetcu R, Currie M, Qureshi R, Mattis P, Lisy K, Mu P-F. Chapter 7: Systematic reviews of etiology and risk . In: Aromataris E, Munn Z (Editors). JBI Manual for Evidence Synthesis. Available online: https://synthesismanual.jbi.global (accessed on
3. Chowdhury, E.; Horrocks, T.; McAteer, G.; Gillanders, D. Examining the impact of androgen deprivation therapy, masculine self-esteem, and psychological flexibility on distress and quality of life in men with prostate cancer. *Psycho-Oncology* **2024**, *33*, 1–10, doi:https://doi.org/10.1002/pon.6277.
4. Jackson, M.D.; Walker, E.; Tulloch-Reid, M.K. Health-related quality of life in long-term Caribbean prostate cancer survivors: comparisons with prostate cancer-free men. *Quality of Life Research* **2022**, *31*, 3391–3401, doi:https://doi.org/10.1007/s11136-022-03202-2.
5. Adam, S.; Koch-Gallenkamp, L.; Bertram, H.; Eberle, A.; Holleczek, B.; Pritzkuleit, R.; Waldeyer-Sauerland, M.; Waldmann, A.; Zeissig, S.R.; Rohrmann, S.; et al. Health-related quality of life in long-term survivors with localised prostate cancer by therapy-Results from a population-based study. *European journal of cancer care* **2019**, *28*, e13076, doi:https://doi.org/10.1111/ecc.13076.
6. Kerleau, C.; Guizard, A.V.; Daubisse-Marliac, L.; Heutte, N.; Mercier, M.; Grosclaude, P.; Joly, F. Long-term quality of life among localised prostate cancer survivors: QALIPRO population-based study. *European Journal of Cancer* **2016**, *63*, 143–153, doi:https://doi.org/10.1016/j.ejca.2016.05.020.
7. Carlsson, S.; Drevin, L.; Loeb, S.; Widmark, A.; Lissbrant, I.F.; Robinson, D.; Johansson, E.; Stattin, P.; Fransson, P. Population-based study of long-term functional outcomes after prostate cancer treatment. *BJU International* **2016**, *117*, E36–E45, doi:https://doi.org/10.1111/bju.13179.
8. Drummond, F.J.; Kinnear, H.; O'Leary, E.; Donnelly; Gavin, A.; Sharp, L. Long-term health-related quality of life of prostate cancer survivors varies by primary treatment. Results from the PiCTure (Prostate Cancer Treatment, your experience) study. *Journal of cancer survivorship : research and practice* **2015**, *9*, 361–372, doi:https://doi.org/10.1007/s11764-014-0419-6.
9. Mols, F.; van de Poll-Franse, L.V.; Vingerhoets, A.J.; Hendrikx, A.; Aaronson, N.K.; Houterman, S.; Coebergh, J.W.; Essink-Bot, M.L. Long-term quality of life among Dutch prostate cancer survivors: results of a population-based study. *Cancer* **2006**, *107*, 2186–2196, doi:https://doi.org/10.1002/cncr.22231.
10. Voerman, B.; Fischer, M.; Visser, A.; Garssen, B.; Van Andel, G.; Bensing, J. Health-related quality of life in Dutch men with prostate cancer. *Journal of Psychosocial Oncology* **2006**, *24*, 49–64, doi:https://doi.org/10.1300/J077v24n02_04.
11. Fowler Jr, F.J.; Collins, M.M.; Corkery, E.W.; Elliott, D.B.; Barry, M.J. The impact of androgen deprivation on quality of life after radical prostatectomy for prostate carcinoma. *Cancer* **2002**, *95*, 287–295, doi:https://doi.org/10.1002/cncr.10656.
12. Al Hussein Al Awamlh, B.; Wallis, C.J.D.; Penson, D.F.; Huang, L.C.; Zhao, Z.; Conwill, R.; Talwar, R.; Morgans, A.K.; Goodman, M.; Hamilton, A.S.; et al. Functional Outcomes after Localized Prostate Cancer Treatment. *JAMA* **2024**, *331*, 302–317, doi:https://doi.org/10.1001/jama.2023.26491.
13. Gaither, T.W.; Kwan, L.; Villatoro, J.; Litwin, M.S. Quality of life in low-income men after surgical castration for metastatic prostate cancer. *Urologic Oncology: Seminars and Original Investigations* **2022**, *40*, 343.e347–343.e314, doi:https://doi.org/10.1016/j.urolonc.2022.04.009.

14. Mazariego, C.G.; Egger, S.; King, M.T.; Juraskova, I.; Woo, H.; Berry, M.; Armstrong, B.K.; Smith, D.P. Fifteen year quality of life outcomes in men with localised prostate cancer: Population based Australian prospective study. *The BMJ* **2020**, *371*, doi:https://doi.org/10.1136/bmj.m3503.
15. Hammerer, P.G.; Wirth, M.P. Health-related quality of life in 536 long-Term prostate cancer survivors after treatment with leuprorelin acetate: A combined retrospective and prospective analysis. *Urologia Internationalis* **2018**, *100*, 72–78, doi:https://doi.org/10.1159/000479434.
16. Punnen, S.; Cowan, J.E.; Chan, J.M.; Carroll, P.R.; Cooperberg, M.R. Long-term health-related quality of life after primary treatment for localized prostate cancer: Results from the CaPSURE registry. *European Urology* **2015**, *68*, 600–608, doi:https://doi.org/10.1016/j.eururo.2014.08.074.
